# Soil as an extended composite phenotype of the microbial metagenome

**DOI:** 10.1038/s41598-020-67631-0

**Published:** 2020-06-30

**Authors:** Andrew L. Neal, Aurélie Bacq-Labreuil, Xiaoxian Zhang, Ian M. Clark, Kevin Coleman, Sacha J. Mooney, Karl Ritz, John W. Crawford

**Affiliations:** 1 Department of Sustainable Agriculture Sciences, Rothamsted Research, North Wyke, EX20 2SB UK; 2 Division of Agriculture and Environmental Science, School of Biosciences, The University of Nottingham, Sutton Bonington Campus, Sutton Bonington, LE12 5RD UK; 3 Department of Sustainable Agriculture Sciences, Rothamsted Research, Harpenden, AL5 2JQ UK; 4 Present Address: Greenback, 4 rue de l’église, 27440 Lisors, France; 5 Present Address: Adam Smith Business School, University of Glasgow, Glasgow, G12 8QQ UK

**Keywords:** Computational biology and bioinformatics, Microbiology, Environmental sciences, Agroecology, Microbial ecology

## Abstract

We use a unique set of terrestrial experiments to demonstrate how soil management practises result in emergence of distinct associations between physical structure and biological functions. These associations have a significant effect on the flux, resilience and efficiency of nutrient delivery to plants (including water). Physical structure, determining the air–water balance in soil as well as transport rates, is influenced by nutrient and physical interventions. Contrasting emergent soil structures exert selective pressures upon the microbiome metagenome. These selective pressures are associated with the quality of organic carbon inputs, the prevalence of anaerobic microsites and delivery of nutrients to microorganisms attached to soil surfaces. This variety results in distinctive gene assemblages characterising each state. The nature of the interactions provide evidence that soil behaves as an extended composite phenotype of the resident microbiome, responsive to the input and turnover of plant-derived organic carbon. We provide new evidence supporting the theory that soil-microbe systems are self-organising states with organic carbon acting as a critical determining parameter. This perspective leads us to propose carbon flux, rather than soil organic carbon content as the critical factor in soil systems, and we present evidence to support this view.

## Introduction

Soil—the basis of terrestrial life on Earth—continues to defy our comprehensive understanding despite the evident catastrophic consequences of mismanagement, such as the North American “Dust Bowl” of the 1930s which was exacerbated by poor stewardship of agricultural soils^1^. Faced with the multiplicity of processes which constitute soil, scientific reductionism has led to studies which have advanced our knowledge of soil’s biological, chemical or physical components predominantly in isolation. However, soil—in common with many biological phenomena—is more appropriately considered a hierarchical assemblage of interacting processes, stabilized and actively maintained at different timescales^2^: soil is processual and not comprehensible based on single-discipline experimentation. Tisdall and Oades’ pioneering conceptual model^3^ linking microbial activity to soil structural development advanced the importance of interaction between biotic and abiotic phenomena in the process of generating soil structural complexity (topology and connectivity).

Soil organic matter (SOM) is the fundamental causative agent generating structural complexity, as it acts to bind mineral particles and colloids together. Plant and animal residues are processed by microbes before joining the SOM pool^4,5^: this step is an important facet of both the Tisdall and Oades model, and its subsequent extension^6^. SOM may take the form of microbial polysaccharidic and proteinaceous exudates as well as cell debris and is chemically structurally diverse^4^; in effect, SOM is a continuum of progressively more extensively oxidized compounds^7^. Much of this SOM is associated with pores of 30–100 μm diameter^8^, scales comparable to the 12–13 μm distances observed in soil between microbial cells^9^. As a result, the effect of microbial processes—metabolism, extracellular degradation of compounds, polymer secretion and cell lysis—on soil structure is particularly evident at the scales < 50 μm responsible for regulating convective and diffusive flow rates, as well as the balance of air and water at any given matric potential^6^. These hierarchical processes exhibit characteristic properties of self-organizing and emergent systems^10,11^.

Such experiential and theoretical approaches are formulating a new understanding of how microbial activity controls soil structure—in effect, how soil should be viewed as an expression of biological process. They also provide evidence supporting a view of soil as a product of genes, manifest through the combined effects of multiple organism phenotypes: in essence, an *extended composite phenotype* (Phillips^12^, after Dawkins^13^). The identifying features of this phenomenon are a strong influence of at least one organism upon the form or structure of a soil environment—termed a *process-form relationship*; demonstrable *synchrony* between the activity of influencing organisms and form development; *selective pressure* arising from form development acting, in Dawkins’ strict sense upon alleles^13,14^ and in Phillips’ broader concept upon soil organisms^12^; which results in *positive feedback* where selective pressure favours alleles (or organisms) associated with the process-form state, manifest as the influence of microbial turnover of SOM upon soil structural development, discussed above.

There is compelling evidence implicating plant-derived organic carbon inputs in the soil extended composite phenotype^15,16^. However, complete description of such a phenotype requires, in turn, a well-developed understanding of the consequences of evolving soil structure for the genetic manifestation of on-going microbial processes—such feedback is necessary for emergence of organisation, observable at the whole-system level in complex biological, chemical and physical systems. Currently, few studies present comprehensive description of the influence of soil structure upon microbial processes, and those that do, typically address only the association of metabolically defined bacterial groups with soil aggregate or particle size, rather than soil structure per se (see Lensi et al.^17^ and Chotte et al.^18^). The principal influence of soil structural complexity is predicted to be on diffusion processes dictating the microenvironments surrounding surface-associated cells^19^. Observation of anaerobic regions of soil aggregates associated with denitrification processes^20^, and the influence of anaerobic microsites in ostensibly oxygen-rich soils upon microbial respiration and carbon compound oxidation rates^21^ provide indirect evidence for such metabolic constraints arising from soil structure. However, this view of soil as an extended composite phenotype requires two specific conditions to be met. The first we term the *Process-Form Condition*, where the biological structures and functions that emerge from interactions between individual genotypes and their microenvironments should result in soil structural changes beyond the scale of individual cells. The second we term the *Allelic Response Condition*, where the process-form interaction should be reflected in significant modification at the level of individual alleles in soil microbiomes (i.e., fundamental changes in gene abundance patterns and whole metabolic pathways) such that alleles that correspond with specific processes are preferentially selected for—extending beyond short-term quantitative changes in specific gene expression profiles.

In this paper, we integrate biological and physical data relating to dynamics of the soil system with mathematical modelling to explore these conditions. This approach is used to interpret results from a unique long-term field-experiment within the context of the proposed view of soil as an extended composite phenotype: linking organic carbon inputs to soil with emergence of key soil structural properties; and describing the gene-level microbiome responses to contrasting emergent soil structural complexity arising from long-term carbon input regimes. The experiment uses the Highfield Ley-Arable Experiment at Rothamsted Research, Harpenden, U.K.

## Results

### Process-form relationships in soil are expressed through fine-scale connected porosity

**Figure 1:**
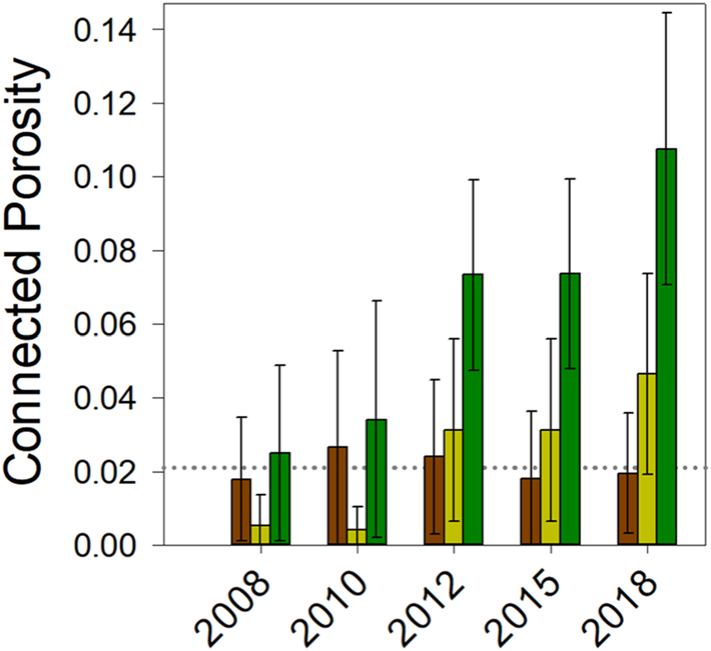
Grassland soils generate connected pore space more rapidly than arable soils. Degraded soil (managed as bare fallow since 1959) developed greater connected micro-porosity following conversion in 2008 to grassland than bare fallow soil converted to arable. The mean and standard error of the mean of connected porosity measured in soil aggregates collected from soil managed continuously as bare fallow (brown), soil converted to arable management (dark yellow) and soil converted to grassland (green) over the ten years following conversion are shown. The dotted line marks the mean connected porosity of continuously managed bare fallow soil over the entire ten-year period.

We first investigated the influence of added organic carbon (C_org_) on the development of soil structural complexity, testing the hypothesis that greater inputs of C_org_ to soil are associated with development of improved soil structure; assessed as the degree of connectedness between pores (connected porosity). The bare fallowed soil used as a starting point for these experiments experienced a demonstrable decline in C_org_^22^ and microbial abundance^23^ over forty-nine years of continuous management. Estimates of C_org_ are approximately 3 g C kg^−1^^24^ and the soil has a significantly reduced total porosity compared to mixed grass sward soils^25^. To assess the influence of newly imposed managements, we followed structure development for a decade (2008–2018) in these degraded soils following conversion to either arable or mixed grass sward. The resulting estimates of soil structure demonstrated clear differences in the development of connected porosity between soil managed continuously as bare fallow and the converted soils (Fig. 1). Testing treatment effects upon connected porosity (square-root transformed to stabilize variances) between 2012 and 2018 inclusive, by analysis of covariance employing time as a covariate, indicated no significant heterogeneity of slopes (*F*_2,75_ = 0.537, *p* = 0.587) due to land management. The resulting equal slopes model indicated a significant effect of management upon connected porosity development (*F*_2,72_ = 26.2, *p* < 0.001). Post hoc pair-wise comparisons indicated that connected porosity generated in grassland soil (mean_adjusted_, 0.079) was significantly greater than in either continuous bare fallow (mean_adjusted_, 0.010) or converted arable (mean_adjusted_, 0.025) soils (smallest difference, *t* = 4.79, *p* < 0.001). A significant difference between connected porosity generated in arable and bare fallow soils was also apparent (*t* = 2.30, *p* = 0.024). No significant differences were detected when the complete 2008–2018 dataset was included, suggesting that significant differences only become apparent after a period of at least 5 years *post* conversion. This represents *prima facie* evidence for an extended composite phenotype, since there is clear synchrony between the establishment and continued growth of plants and development of connected porosity, representing a process-form relationship.

**Figure 2:**
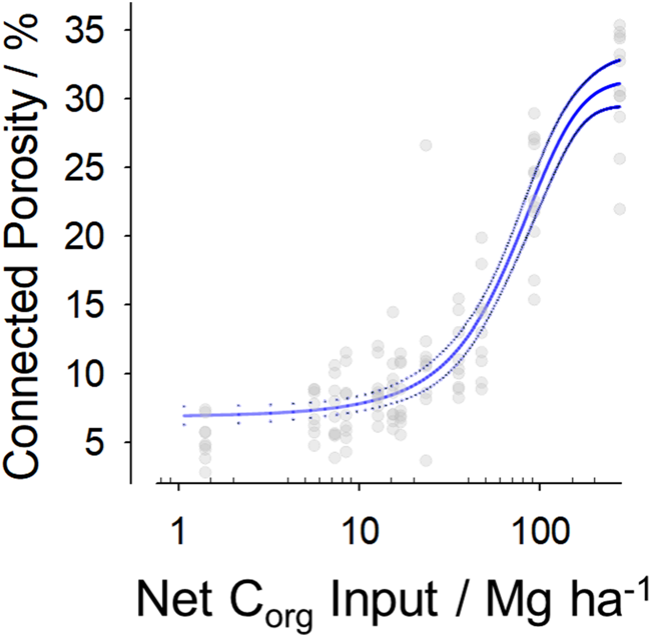
Soil process-form relationships reflect biotic organic carbon inputs and turnover. The connected pore space in degraded soils converted after 49 years of bare fallow management to either arable or grassland increases in association with the net input of organic carbon (C_org_). Soils managed continuously as either arable (for 66 years) or grassland (for > 175 years) which have each accumulated over 100 Mg ha^−1^ of C_org_ over their history follow this trend. The relationship is described by an asymptotic function; the resulting fit (solid line) is shown, together with the upper and lower 95% confidence intervals of the fit (dotted lines). *R*^2^ = 0.85.

To test the potential role of C_org_ in the observed relationship, we compared connected porosity development with soil C_org_ inputs. We modelled changes in C_org_ content (see “Methods” for details) in bare fallow soil from inception of the experiment in 1949 up to the year before conversion, 2007. From 2008, C_org_ changes were modelled for each year, based upon continued bare fallow management or management as either winter wheat based arable or mixed grass sward. Continuously managed arable and grassland soils were also modelled in the same manner but using their respective starting dates. The extremes of the cumulative C_org_ input-connected porosity relationship (Fig. 2) are derived from consistently managed soils; bare fallow being associated with the lowest net C_org_ input and connected porosity, and arable and grassland soils being associated with the second highest and highest C_org_ input and connected porosity, respectively. Modelled C_org_ inputs and measured connected porosity for soils converted to both arable and grassland for each of the ten years between 2008 and 2018 is distributed between the consistently managed bare fallow and arable soils data. We assumed that C_org_ in soils managed as grassland since 1838 represented the maximum which could be stored; this and the Akaike information criterion was used to guide selection of a sigmoidal function. There was a clear non-linear relationship between C_org_ inputs to soil and connected porosity, with all converted and continuously managed soils following the same trend (Fig. 2). This establishes that process-form relationships can be explained in terms associated with biotic C_org_ inputs and turnover in soil.

### Contrasting long-term soil management results in different process-form states

Although the converted soils provided compelling evidence for structural development contemporaneous with the establishment and development of plant populations (albeit that arable soils are subject to external processes such as tillage and fertilization), the relatively short time span did not allow comparison of maximal differences in structural development, or evaluation of the potential for any resulting selective pressures to influence the representation of specific genes within the soils. Soils which had been under continuous management, at the time of sampling, as bare fallow for 52 years, arable for 62 years and mixed grass swards for over 170 years presented an opportunity to test the hypothesis that established process-form relationships result in selection of organisms or genes, the fitness of which is suited to each particular soil biotope.

We have already determined that the continuously managed grassland and arable soils have significantly greater total porosity, a wider range of pore sizes and greater pore connectivity than continuously managed bare fallow soil^25^. Here we extend these findings, generating detailed information concerning pore topology and connectivity since they exert a strong influence upon diffusive flow in porous materials^26^, and modelling the effect of the observed pore networks upon diffusion processes within the soils. Euler connectivity functions [*χ*(*d*)/*V*, see “Methods” section for calculation] for each soil are shown in Supplementary Fig. 1. For connected pores *χ*(*d*)/*V* < 0, the value is positive for unconnected pores. *χ*(*d*)/*V* = 0 represents a critical threshold diameter (*d*_crit_) describing the maximum pore throat size of connected pores controlling hydraulic conductivity^27^. Mean estimates (± standard error) of *d*_crit_ were 9.7 ± 0.37 µm for grassland soils, 7.2 ± 0.26 µm for arable soils, and 3.1 ± 0.76 µm for bare fallowed soils. There was a significant effect of soil management upon *d*_crit_ (*F*_2,6_ = 42.3, *p* < 0.001) and each mean was significantly different from all others (smallest difference, grassland versus arable, *Q* = 4.99, *p* = 0.029). Topology-related parameters derived from X-ray computed tomography of aggregates (Table 1) showed a consistent trend of greater parameter estimates in grassland soils than degraded bare fallow soils. We chose porosity and *d*_crit_ as measures of pore topology since their implications are readily defined and they are of direct relevance to cells within the soil matrix because of their influence upon advective and diffusional processes. Porosity measurements from X-ray CT were used to derive diffusion coefficients for solutes within saturated soil aggregates, relative to unconstrained solute diffusion in water (*D*/*D*_0_, see “Methods” for details). For grassland soils, mean *D*/*D*_0_ was determined at 0.399 ± 0.014, 0.285 ± 0.009 for arable and 0.161 ± 0.001 for bare fallow. These estimates were significantly different (*F*_2,70_ = 106.4, *p* < 0.001). Normalised diffusion coefficients for each treatment were all significantly different from each other (*p* < 0.001 for all comparisons).

**Table 1:** Topology-related parameters derived from binary images generated from X-ray computed tomography of aggregates from Highfield soils.

	Porosity/%	Permeability/mm^2^	Connectivity/µm^−3^	Pore surface density/µm^2^ µm^−3^	*d*_crit_/µm	Pore neck size/µm
Grassland (*n* = 14)	31.1 ± 1.18	1.13 ± 0.310	− 0.206 ± 0.025	0.088 ± 0.003	9.74 ± 0.37	11.19 ± 0.34
Arable (*n* = 14)	23.4 ± 1.22	0.62 ± 0.154	− 0.236 ± 0.033	0.092 ± 0.004	7.17 ± 0.26	8.79 ± 0.48
Bare fallow (*n* = 9)	15.0 ± 2.21	0.55 ± 0.339	− 0.018 ± 0.080	0.059 ± 0.010	3.10 ± 0.76	4.72 ± 0.95

The mean and standard error of each parameter is shown.

**Figure 3:**
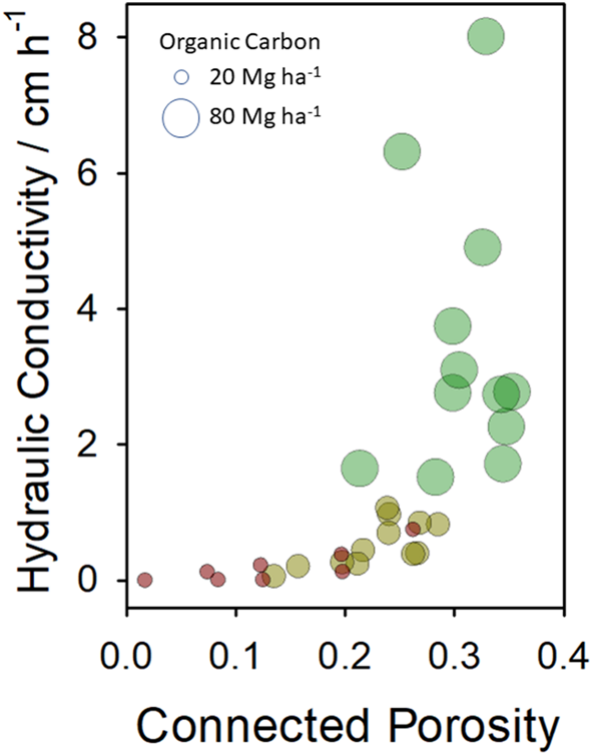
Contrasting long-term soil management results in quantitatively different process-form states. Soils are described by a combination of the connectivity of pore space, established from X-ray connected porosity (CT) and modelled hydraulic conductivity—a measure of capacity, representing the maximum potential movement of resources through pore networks to organisms. Grassland soils (green data points) are characterized as having high pore connectivity and hydraulic conductivity and are associated with the greatest stocks of C_org_. In contrast, degraded bare fallow soils (brown data points) are associated with extremely limited connected porosity and hydraulic conductivity and the lowest stocks of C_org_. Arable soil (dark yellow) is intermediate between these two extremes. Data point size is proportional to C_org_ (Mg ha^−1^) in each soil, the extremes of which are shown in the key.

**Figure 4:**
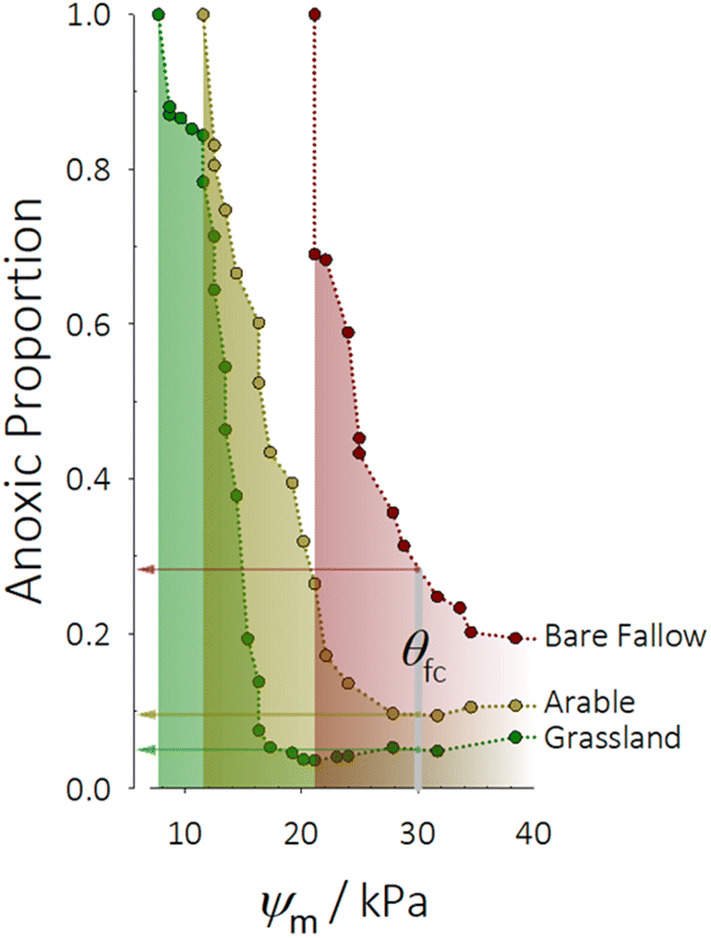
Process-form states control anoxia within soil. Low-C_org_, low-connected porosity soil contains much larger volumes of anoxic microsites than high-C_org_, high-connected porosity soil. Across a range of matric potential (*ψ*_m_), the predicted volume of anoxic sites is consistently larger in degraded bare fallowed soil than arable or grassland. At field capacity (*θ*_fc_), approximately 30% of degraded soil is anoxic, falling to 5% in grassland soil. At 21 kPa degraded soil is completely anoxic while the volume remains between 4–5% in grassland soil. In arable soil 10% of the soil volume is predicted to be anoxic at *θ*_fc_—double that in grassland.

Direct measurement of pore network topology and modelling of the consequences for diffusion demonstrate that different long-term soil management results in quantitatively different process-form states. We also used estimates of pore network topology to model the hydrodynamic behaviour of the pore networks under saturated conditions, estimating hydraulic conductivity as a function of connected porosity. This measures the dynamical state of the pore space and the maximum potential flow rate at which resources can move through the networks—effectively the capacity for flux within the soil pore space. Figure 3 shows the combined effects of soil C_org_ and connected porosity upon the predicted hydraulic conductivity of soils. Combined direct measurements and modelling indicate a power law relationship between connected porosity and conductivity and that C_org_ is associated with these changes. Regions of this relationship correspond to the process-form states of continuously managed bare fallow, arable and grassland soils. The fraction of anoxic volume characterising each process-form state was estimated using a multi-phase lattice-Boltzmann approach^28,29^, described in detail in the “Methods” section. The results (Fig. 4) indicate that the predicted anoxic fraction is significantly lower in grassland soil, compared with arable and bare fallowed soils, the latter is predicted to have the highest fraction of anoxic volume at all matric potentials (moisture contents).

Microorganisms in each land management-associated process-form state are likely to experience markedly different hydraulic environments, particularly in degraded bare fallow soils where reduced delivery of dissolved nutrients and O_2_ is predicted compared to grassland soils. This is a direct result of constraints placed upon diffusive flow by reduced connected porosity and *d*_crit_ resulting from different biotic C_org_ inputs and turnover. These constraints are likely to exert significant selective pressures in soil microbiomes which should be reflected in changes in the assemblages of organisms or alleles in the different process-form states. To test this hypothesis, we generated shotgun metagenome sequence datasets from nucleic acids extracted directly from the different soils. These were analysed to determine whether any observed differences in phylogenetic community assemblages or in gene abundance were directly attributable to the differences in porosity or *d*_crit_ described above.

### Process-form states in soil do not exert selective pressure at the organismal level

**Figure 5:**
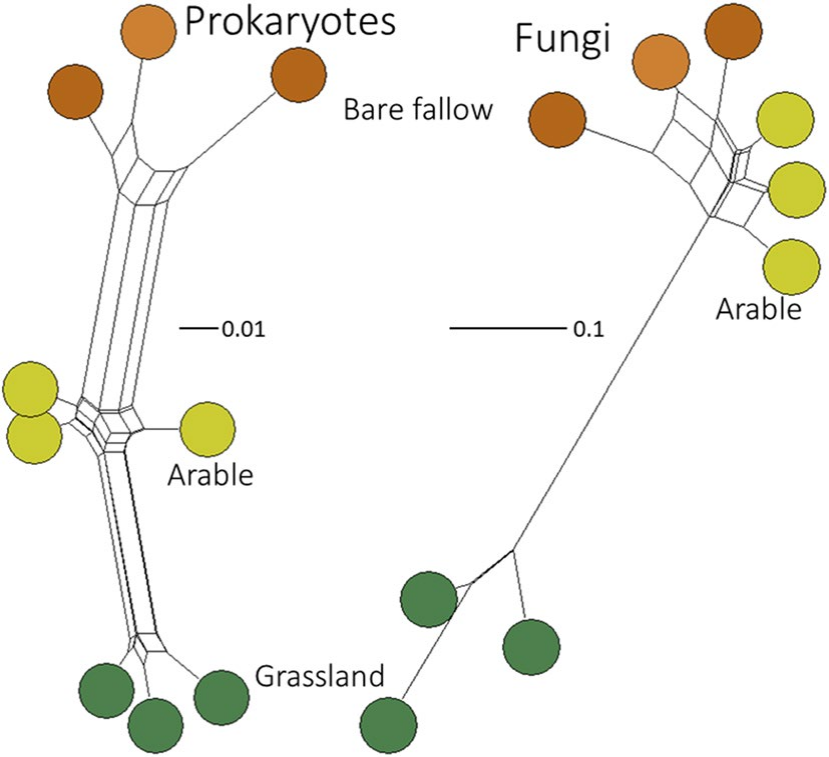
Grassland, arable and bare fallowed soil microbial community β-diversity. Neighbour-Net networks of prokaryotic and fungal community profiles from the three soil managements based on weighted UniFrac distance. Prokaryotic community assemblages were significantly phylogenetically different between all three managements; for fungi there was no difference between arable and bare fallow soil assemblages, which were both significantly different from grassland assemblages.

Chao-1 lower bound estimates of Prokaryote OTU richness (*S*_Chao1_) for each land management ranged from 562 to 578 (mean 570) for grassland, 530–547 (mean 540) for arable, and 482–542 (mean 513) for bare fallow soils. There was a significant effect of soil treatment upon *S*_Chao1_ (*F*_2,6_ = 7.6, *p* = 0.023), the difference between grassland and bare fallow mean richness was significant (*Q* = 5.49, *p* = 0.019). There was no significant difference between arable and grassland or arable and bare fallowed soils. Grassland soils also exhibited the largest Fungal OTU richness, range 35–44 (mean 39) compared to either arable (range 19–27, mean 24) or bare fallowed (range 17–27, mean 23) soils. There was again a significant treatment effect upon *S*_Chao1_ (*F*_2,6_ = 11.8, *p* = 0.008) and pair-wise comparison indicated grassland was significantly more rich in fungal OTUs than either arable or bare fallowed soils (smallest difference, *Q* = 5.68, *p* = 0.017), but there was no difference between arable and bare fallowed soils. Weighted UniFrac distance-based comparison of β-diversity (Fig. 5) indicated significant effects of soil management upon both prokaryotic (PERMANOVA, *pseudo-F*_2,6_ = 15.5, *p*_perm_ < 0.0001) and fungal (*pseudo-F*_2,6_ = 19.0, *p*_perm_ = 0.0032) community structures. Prokaryote communities were significantly different between all three treatments (smallest difference, *pseudo-t* = 2.9, *p*_MC_ < 0.0001) but fungal communities in arable and bare fallowed soils did not differ (*pseudo-t* = 1.7, *p*_MC_ = 0.111); both were significantly different from the grassland community (smallest difference, *pseudo-t* = 5.0, *p*_MC_ = 0.0015). Inspection of individual fungal OTU abundance indicated that this was due to several OTUs, including *Rhizophagus irregularis* (formerly *Glomus intraradices*) and other Glomeromycetes, Agaricomycetidae, Onygenales, Eurotiomycetidae, Aspergillaceae and Atheliaceae, being abundant in grassland soils but not detected in either arable or bare fallowed soils: arbuscular mycorrhizal *R. irregularis*, for example, had a mean abundance in grassland soils of 3.5 × 10^5^, but was not detected in the other soils. This large, qualitative, difference between the soils is consistent with the effect of soil tillage^30,31^ upon fungal communities and cannot be interpreted as a response to selective pressures arising from different process-form states. Since prokaryotes appeared to be less sensitive to the effects of tillage than fungi, the effects of soil management upon prokaryotic communities were studied in detail.

**Figure 6:**
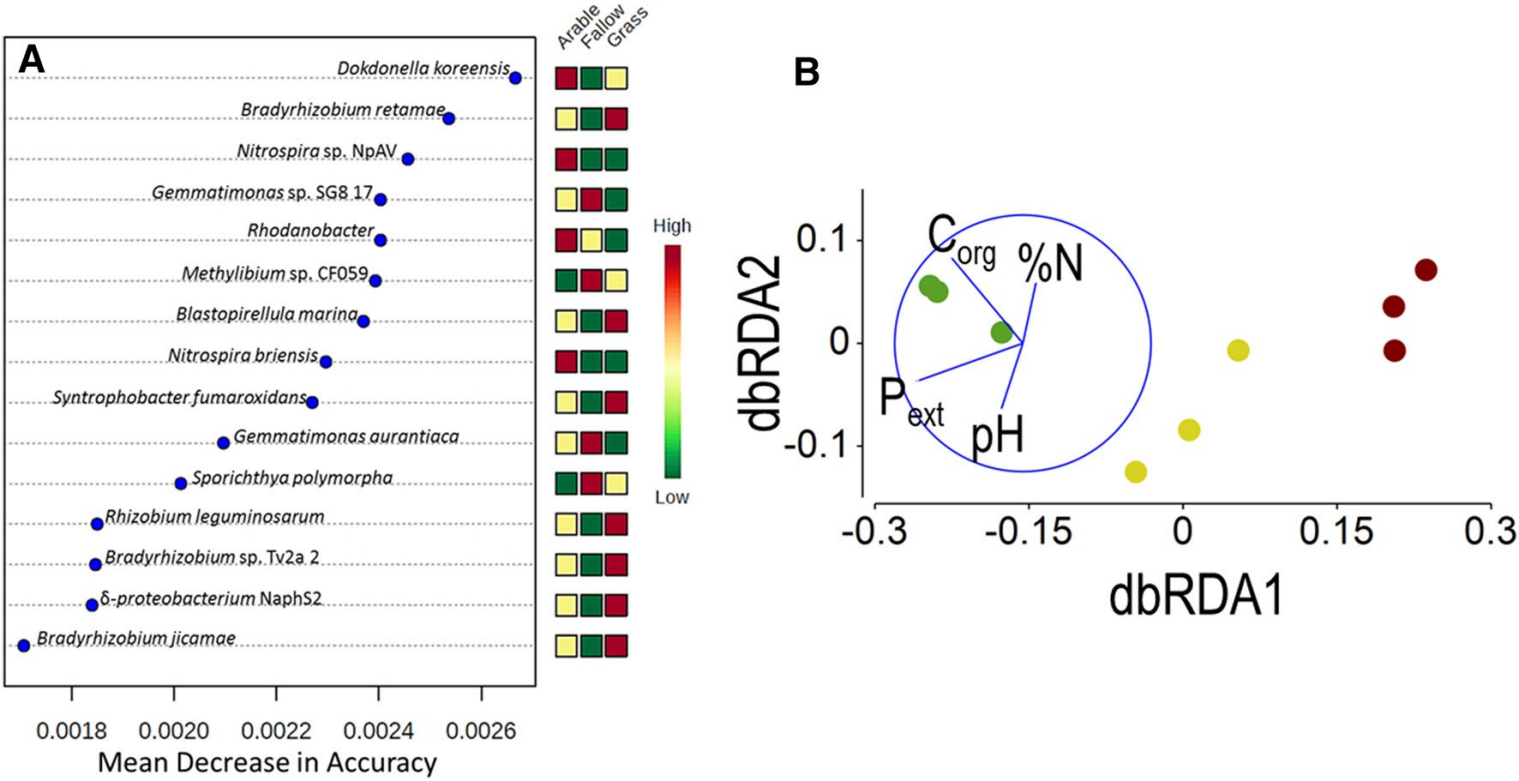
Taxonomy- and phylogeny-based community responses to land management. (**A**) Predictive modelling using a supervised Random Forest algorithm identified the fifteen OTUs that were most discriminatory between the different soils, based upon the mean decrease in model accuracy of a leave-one-out cross-validation procedure. (**B**) Management-conditional distance-based redundancy analysis (dbRDA) of chemical edaphic factors and 16S rRNA gene-based assessment of microbiomes associated with the Highfield Ley-Arable experiment using Kantorovich–Rubinstein phylogenetic distances calculated from placement of homologous metagenome reads on the 16S rRNA gene reference phylogenetic tree. Data points represent individual replicate plots of grassland (green), arable (yellow) and bare fallow (brown) soils. Environmental factors (pH, NaOH-EDTA extractable P [P_ext_], C_org_ and % organic N [%N]) were determined by distance-based linear modelling as the most parsimonious combination of variables to model the multivariate data and are represented as vectors, increasing in the direction of the vector: vector length indicates the degree of partial correlation of each environmental variable with the dbRDA axes. The circle has an arbitrary origin and radius of *r* = 1. dbRDA axis 1 accounted for 83.95% of variation accounted for by the model (74.71% of total variation) and dbRDA axis 2 accounted for 9.96% of variation accounted for by the model (8.87% of total variation). *R*^2^ = 0.8899. The corresponding unconstrained PCoA ordination is shown in Supplementary Fig. 2. See text for a detailed description of the analysis.

Random Forest machine learning classification of biomarkers (Fig. 6A) indicated prokaryotic communities in grassland soils were characterized by Rhizobiaceae including *Bradyrhizobium* spp. and *Rhizobium leguminosarum* as well as the planctomycete *Blastopirellula*. At the other extreme, taxa characteristic of degraded, low input bare fallow soils were *Gemmatimonas* spp., an organism related to the aromatic compound degrader *Methylibium* and the actinomycete *Sporichthya*. The influence of nitrogenous fertilization was evident in the organisms identified as characteristic of arable soils; nitrite-oxidizing *Nitrospira* spp. were particularly characteristic of these soils together with the denitrifying *Rhodanobacter* and *Dokdonella koreensis*^32^.

16S rRNA gene-conditional phylogenetic diversity based upon placement of exact sequence variants for each treatment was compared using Kantorovich-Rubinstein (KR) distance metrics. PERMANOVA identified a significant effect of treatment (*pseudo-F*_2,6_ = 17.9, *p*_perm_ < 0.0001) and all post hoc comparisons were significantly different (smallest difference: bare fallow *vs*. arable, *pseudo-t* = 3.2, *p*_MC_ = 0.0018) consistent with the weighted UniFrac approach described above. Principal coordinates analysis (PCoA) was used to present an unconstrained view of differences in 16S rRNA gene-conditional microbiome assemblages (Supplementary Fig. 2) using KR distance. The first two principal coordinates separated treatments clearly, the ordination accounting for 89% of total variation across the two axes. Distance-based linear modelling (distLM) was used to describe the relationship between the 16S rRNA gene-conditional phylogenetic community structure and edaphic variables shown in Table 2. All combinations of variables were considered: the most parsimonious model, identified using Bayesian information criterion (BIC), was a combination of the chemical factors pH, soil C_org_, soil organic nitrogen (%N) and NaOH-EDTA extractable phosphorus (P_ext_). Distance-based redundancy analysis (dbRDA) indicated the model accounted for 84% of total variation on the two axes (Fig. 6B). Separation of treatments on dbRDA axis 1 was associated most highly with P_ext_ (*r* = − 0.81; marginal test, *pseudo-F* = 7.4, *p*_perm_ = 0.013) and C_org_ (*r* = − 0.53; marginal test, *pseudo-F* = 12.2, *p*_perm_ = 0.0035), both greatest in grassland soils and least in bare fallowed soils. The second axis was most highly associated with C_org_ (*r* = − 0.88) and %N (*r* = 0.41; marginal test, *pseudo-F* = 11.3, *p*_perm_ = 0.004). Using these four chemical parameters to model the distribution, addition of neither porosity (sequential test, *pseudo-F* = 0.7, *p*_perm_ = 0.565) nor *d*_crit_ (sequential test, *pseudo-F* = 0.5, *p*_perm_ = 0.691) accounted for a significant amount of additional variation.

**Table 2:** Summary of chemical data of Highfield Ley-Arable experiment soils.

	pH (H_2_O)^a^/−log(g[H^+^]L^−1^)	Organic carbon^a^/mg g^−1^ soil	Total nitrogen^a^/µg g^−1^ soil	NaOH-EDTA extractable phosphorus^b^/µg g^−1^ soil
Grassland	6.2 ± 0.13	3.72 ± 0.44	340 ± 39.0	661.7 ± 31.3
Arable	5.8 ± 0.11	1.85 ± 0.06	190 ± 5.08	517.0 ± 12.6
Bare fallow	5.3 ± 0.19	1.07 ± 0.10	110 ± 6.71	235.0 ± 3.8

The mean and standard error of the mean are shown (*n* = 3).

^a^Gregory et al.^22^.

^b^Neal et al.^68^.

Although there are clear qualitative and phylogenetic differences between prokaryote assemblages associated with each soil, distLM suggests that these differences are adequately described by chemical edaphic parameters. They are therefore unlikely to be due to selective pressure arising from the respective process-form states directly. Instead, assemblage differences are likely to reflect organism traits: for example, Gemmatimonadetes are common in soil and show adaptation to low soil moisture^33^, so identification of *Gemmatimonas* as characteristic of bare fallow soil is likely to reflect the fact that direct isolation experienced by these soils renders them much drier than the other soils; nitrogenous fertilization of arable soils is reflected by the organisms identified as characteristic of these soils to be either nitrite-oxidisers or denitrifiers; and while identification of Rhizobiaceae as characteristic of the mixed swards of grassland soils suggests association with legumes—and therefore possibly responsive to selection pressure exerted by the plant population, *Bradyrhizobium* spp. in these soils lack genes and gene clusters for symbiosis and nitrogen fixation^34^.

### Process-form states in soil exert selective pressure at the level of alleles

**Figure 7:**
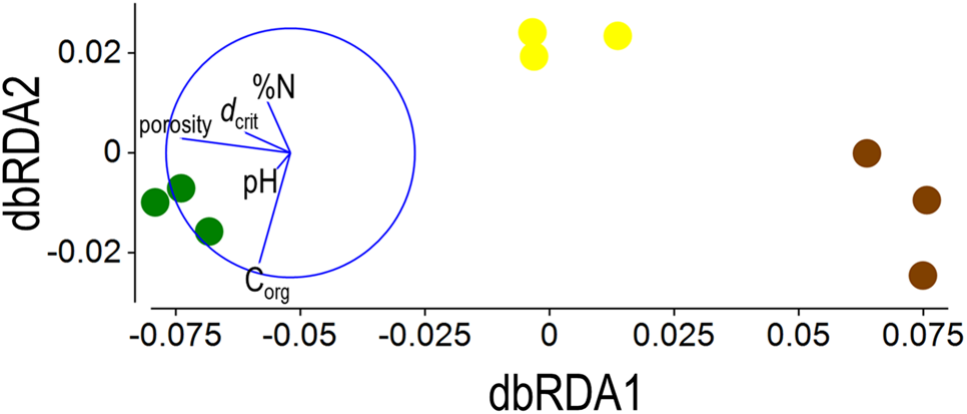
Function-based community responses to process-form states. Management-conditional dbRDA of chemical and physical edaphic factors and function-based assessment of genes associated with the Highfield Ley-Arable experiment. Square root transformed KEGG ortholog abundances were used to calculate Hellinger distances between the nine samples. Data points represent individual replicate plots of grassland (green), arable (yellow) and bare fallow (brown) soils. Environmental factors (pH, C_org_, % organic N [%N], porosity and *d*_crit_) were selected by distLM as the most parsimonious combination of variables to model the multivariate data and are represented as vectors, increasing in the direction of the vector: vector length indicates the degree of partial correlation of each environmental variable with the dbRDA axes. The circle has an arbitrary origin and radius of *r* = 1. dbRDA axis 1 accounted for 88.40% of variation accounted for by the model (83.21% of total variation) and dbRDA axis 2 accounted for 7.17% of variation accounted for by the model (6.75% of total variation). *R*^2^ = 0.9414. The corresponding PCoA ordination is shown in Supplementary Fig. 8. See text for a detailed description of the analysis.

A total of 1,197 KEGG orthologs were identified as having significantly different abundance between the soils (selected orthologs are presented in detail in Supplementary Figs. 3–7). We adopted a similar approach to analysing the effects of soil management upon microbiome genetic variation, determined by binning reads to KEGG orthologs, as for the effect upon community assemblage, described above. Multivariate ortholog analysis was based on Hellinger distance, calculated from square root-transformed ortholog abundance. PERMANOVA identified a significant effect of land use upon ortholog assemblage (*pseudo-F*_2,6_ = 26.8, *p*_perm_ < 0.0001) and all post hoc comparisons were significantly different (smallest difference: arable *vs*. bare fallow, *pseudo-t* = 3.6, *p*_MC_ = 0.0006). PCoA separated each land use, the first two axes accounting for 91% of total variation (Supplementary Fig. 8). The most parsimonious model identified by distLM and BIC included a combination of both chemical and physical edaphic variables; namely pH, C_org_, %N, porosity and *d*_crit_. dbRDA (Fig. 7) showed clear separation between the treatments on dbRDA axis 1. The edaphic variables associated most highly with this axis were both physical parameters; porosity (*r* = − 0.87; marginal test, *pseudo-F* = 24.7, *p*_perm_ = 0.0009) and *d*_crit_ (*r* = − 0.36; marginal test, *pseudo-F* = 15.2, *p*_perm_ = 0.0019). Both variables were greatest in grassland soil and least in bare fallowed soil. The treatments showed little separation on the second axis. Edaphic variables associated most highly with this second axis were chemical, C_org_ (*r* = − 0.88; marginal test, *pseudo-F* = 17.3, *p*_perm_ = 0.0002), %N (*r* = 0.47; marginal test, *pseudo-F* = 0.42, *p*_perm_ = 0.633) and pH (*r* = 0.41; marginal test, *pseudo-F* = 13.9, *p*_perm_ = 0.0038). Using these edaphic parameters to model the distribution, addition of P_ext_ (sequential test, *pseudo-F* = 0.97, *p*_perm_ = 0.484) did not account for a significant amount of additional variation.

**Figure 8:**
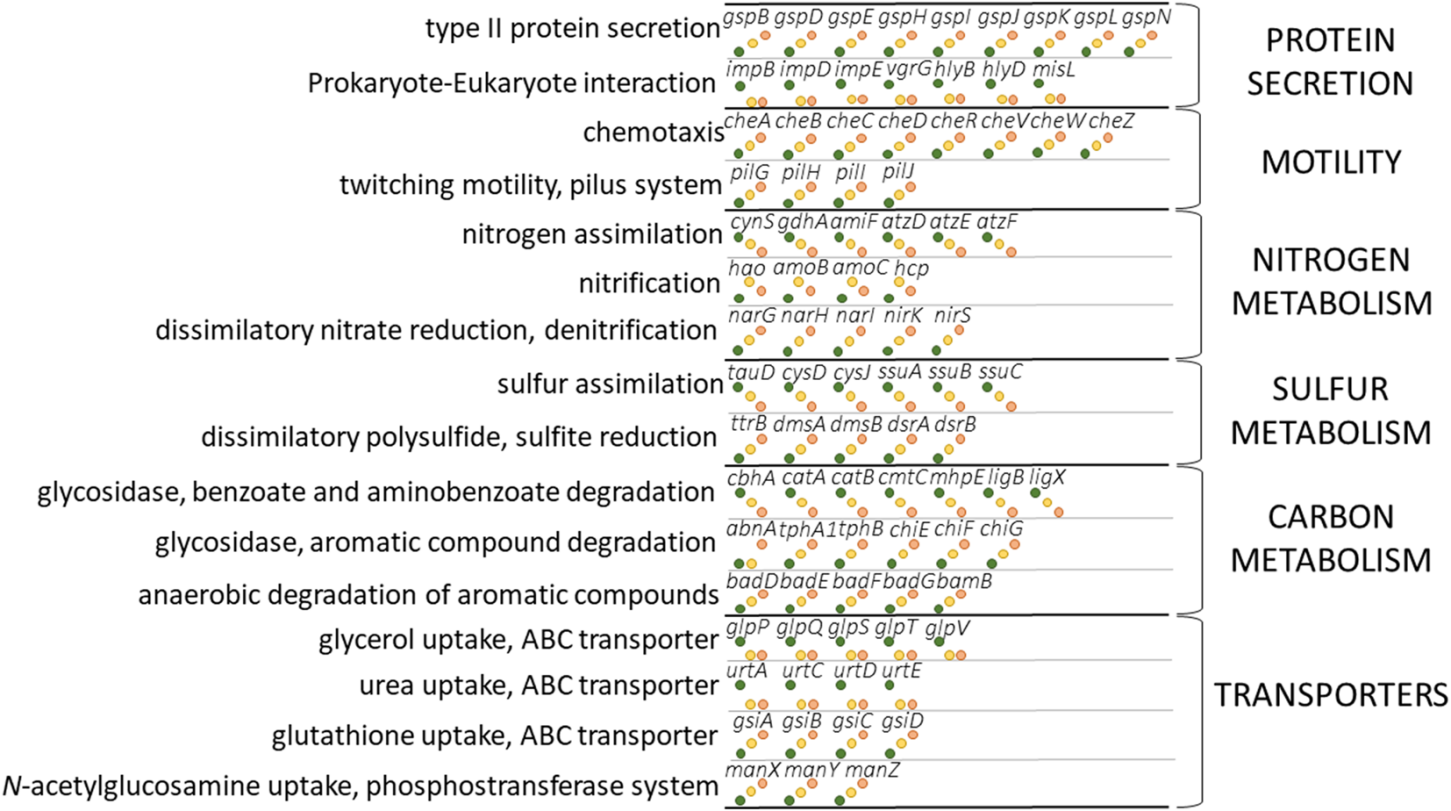
Schematic representation of the relative abundance of genes for which significant differences between process-form states was determined. The central column indicates the general trend in relative abundance for genes grouped according to specific functions. Grassland gene abundance is represented as green points, arable gene abundance as yellow points and bare fallow gene abundance as brown points: each specific function is described in the left-hand column; specific functions are organized into higher-level KEGG ontologies, shown in the right-hand column. Absolute abundances for each gene and associated significance (*p*) and positive false discovery rate (*q*) of the difference in abundance between the three treatments are shown in Supplementary Figs. 3–7.

This analysis presents clear evidence for a large and direct influence of process-form state upon gene assemblages. The topological parameters shown to be so influential upon gene distributions are both fundamental properties of pore networks which we have shown to be sensitive to biotic C_org_ inputs and turnover in soil (Figs. 2, 3). We have also demonstrated that they exert a dominant influence upon hydrodynamic conductance of the pore network and the potential for anaerobic sites across a range of soil matric potential (Figs. 3, 4). However, the analysis cannot demonstrate preferential selection of genes dependent upon their fitness within each process-form state. To test whether the differences in gene abundance could be due to selection pressures arising from different plant inputs and emergent soil structural properties, we characterised the genes shown to be sensitive to the different land managements. Consideration of changes in individual gene abundance indicated clear shifts in both cellular behaviour and metabolic potential (Fig. 8). For cell behaviour, there were a number of genes associated with protein secretion, among them *impB*, *impD*, *impE* and *vgrB* associated with bacterial type VI protein secretion systems, *hylB* and *hylD* associated with type I protein secretion systems and the autotransporter gene *misL*: all typify bacterial-bacterial and bacterial-Eukaryotic interactions and were more abundant in grassland soils. Genes associated with type II protein secretion systems (T2SS) were more abundant in arable and bare fallow soils, suggesting a greater reliance upon exoenzymes in these soils. Consistent with this latter observation, several genes coding for exoenzymes were more abundant in these soils, including *abnA* (glucosyl hydrolase [GH] family 43 endo-arabinanase), *chiE* (GH family 18 chitinase) and *chiF* and *chiG* (both GH family 19 chitinases) associated with carbohydrate metabolism, and *dmsA* and *dmsB* (dimethyl sulfoxide reductase) associated with sulfur metabolism. Genes coding for chemotaxis and twitching motility were also more abundant in arable, and particularly bare fallowed soils compared to grassland soil.

The increase in abundance of *dmsAB* was also part of a general trend of an increase in genes associated with dissimilatory anaerobic metabolism of nitrogen and sulfur in arable and bare fallowed soils combined with reductions in genes associated with assimilatory pathways. Nitrification-associated genes were most abundant in arable soils, and genes associated with dissimilatory reduction of nitrate and sulfate most abundant in bare fallowed soils. There was also an increase in genes associated with anaerobic degradation of aromatic compounds in arable and bare fallowed soil. Transport pathways also differed between land use with genes associated with ATP-binding cassette (ABC) transporter pathways of glycerol and urea being most abundant in grassland soil and least abundant in bare fallowed soil, while genes associated with the ABC transport pathway for glutathione and the *N*-acetylglucosamine phosphotransferase pathway exhibited the opposite trend.

These genetic shifts were related to nutrient status and, saliently, changes in soil structure (in this case pore topology) controlling gaseous and nutrient diffusion. They present direct evidence for genetic selection of genes and pathways based upon fitness under the different process-form states. The increase in genes associated with less efficient anaerobic processes in arable and bare fallowed soil can be considered a response to reduced diffusion of O_2_ in these progressively more poorly connected pore networks. Other responses, such as the increase in gene abundance for chemotaxis and protein secretion, may also be responses to reduced diffusion of soluble nutrients, and hence a requirement to search out nutrients, or avoidance of anaerobic niches within the soil. Microbial community structure is often considered as a balance of cooperative behaviours between individuals, mediated by “public goods” or soluble nutrients arising from leaky processes (nutrients which are lost through the outer membrane or released by cell lysis) or the activity of exoenzymes^35,36^. Producers of public goods support populations of “cheaters” which exploit goods without contributing to them. In well-mixed systems, cheaters maintain a competitive advantage over producers, but this advantage is lost in structured environments where diffusive constraints are manifest^37^. In this context, the increase of T2SS and arabinanase and chitinase exoenzyme genes in arable and bare fallowed soils may be a response to both qualitative changes to organic inputs, and reduced delivery of soluble nutrients by advective flow and diffusion to cheaters, and thus an increase in abundance of producer organisms. Additionally, the reduced diffusive processes predicted for arable and particularly bare fallowed soil may result in an increased efficiency of exoenzymes since reduced diffusion allows for a greater accumulation of product near producer organisms^38^. Thus, production of exoenzymes, and cell motility as a searching or avoidance behaviour provide adaptations in response to spatially constrained circumstances arising from reduced pore connectivity as a result of reduced C_org_ inputs in arable and bare fallowed soils.

## Discussion

We have presented data consistent with the conditions which should be met if soil is an extended composite microbial phenotype: the emergent physical states are organised at several orders of magnitude above the scale of individual microbes; different physical states are associated with different genetic states at the level of individual alleles rather than organisms. The data also demonstrates that both physical and biotic states of the system can be manipulated by nutritional interventions, particularly relating to the flux of energy through the system.

The data do not prove the associations are causal, however comparison with simpler systems that are amenable to deeper theoretical analysis and direct manipulation provides additional evidence of a causal feedback between allelic abundance, process and form. Specifically, the soil-microbe system exhibits behaviour seen in physical systems that display spontaneous (and endogenously driven) emergence of large-scale self-organisation as a result of such feedback. In a subset of such systems, there is a critical point at which the state of the system changes discontinuously (a *phase transition*) with continuous change in one of the system parameters. The rate of change of the system with respect to that parameter is characterised by a power law close to the phase transition, reflecting the emergence of coherence across a wide range of scales (often referred to as fractal scaling). In Fig. 3 we observe such behaviour between connected porosity and hydraulic conductivity. The state of the soil system changes from one with a disconnected pore space to one with a connected pore space where C_org_ (energy) flux is a critical parameter. There is a power law relation between conductivity and porosity, consistent with the emergence of large-scale spatial coherence in soil structure at a critical value of C_org_ flux. In this sense, soil displays many of the properties of self-organising systems^39^. Results presented here provide further evidence for a causal feedback between allelic abundance, process and form. We have previously posited a mechanism for this in soil and shown how soils with and without plants are capable of spontaneously generating emergent structures at important scales^6^ compared with sterile soils, which do not. This interpretation predicts that soils which are more self-organising will be more metabolically active in any given situation than a soil where the interaction between biological process and form is weak or non-existent. We see that, after a minimum of 52 years, each soil in our study is a different expression of its multiple biotic components; a phenomenon termed an extended composite phenotype^12^.

With plants present, such as land managed as long-term mixed grass sward, the extended composite phenotype has an increased capacity to store water and soluble nutrients, a property which may confer a degree of resilience to the soil–plant–microbe system during periods of low rainfall or nutritional inputs. Independent analysis of these same soils has demonstrated greater water storage capacity in the grassland soil^40^. In addition, the more extensive and more connected pore network selects for assimilatory, and against dissimilatory, processes by permitting greater flux of O_2_ through the system: it thus improves the efficiency of metabolic processes and C_org_ conversion into biomass while reducing potential losses of plant nutrients arising from leaching or emission to the atmosphere. Thus, the extended phenotype interacts with plants to increase the flux, resilience and efficiency of nutrient transport to plants (including water).

The finding that soil under grassland management has significantly higher capacity, efficiency and resilience compared with arable or bare fallowed management is associated with greater C_org_ inputs and turnover. Furthermore, the rate of recovery of degraded soil is also linked to stocks and flows of C_org_ (Fig. 1). Our experiments cannot distinguish between C_org_ flux or storage as the dominant mechanism supporting improved soil function. However, interpreting results in terms of soil remodelling through self-organizing processes, we predict that the biophysical state of soil and rate of change of that state will both be related to cumulative metabolic activity. Our data are consistent with recovery rate being limited by cumulative soil metabolism: soil C_org_ content acts as a diagnostic for this. This raises the important questions of what limits soil metabolism and incorporation of C_org_ in soil^41^, and how it can be manipulated in each context to maximise the rate of soil recovery. We know both anaerobic niches and physical dislocation of microbes from resources result from low pore connectivity, and both significantly limit microbial metabolism. We also know soil recovery is associated with more voluminous and better-connected pore space and significantly lower levels of anaerobic respiration. We speculate that the rate-limiting factor in recovery of degraded soil is the process of microbially-mediated micro-structure remodelling, and that this is soil texture dependent^25^. Sandy-textured soil would be less able to recover compared to soils with higher fractions of silt and clay, where remodelling fine-scale structure is inherently more feasible due to a greater proportion of “raw materials” to enable such fine-scale architecture to be manifest. It is also likely to be dependent on the quality and quantity of organic inputs to soil, especially in relation to the latent energy contained in them. This is apparent in our data, though we are not able to distinguish the relative importance of each.

Tillage is known to contribute to decreases in soil C_org_, and the most effective recovery rate and highest metabolizing end-state in our data was achieved with management under grassland without tillage. Tillage has the effect of significantly changing the distribution of microenvironments in soil through increased aeration and exposure of previously physically protected prey organisms and soil C_org_. This results in the immediate release of physical and chemical constraints on metabolism and therefore to loss of soil C_org_. More importantly, rearrangement of microenvironments—i.e. within and between soil macro- and micro-aggregates—will have the effect of “re-setting” microbial remodelling of soil microarchitecture, slowing down establishment of connected pore space and longer-term cumulative metabolism.

This new interpretation of the role of nutritional and physical management of soil is a step towards a more general theory of soil. Such a theory is needed as a framework upon which to synthesize data and knowledge on biological, chemical and physical properties of soil that are typically studied in isolation. Theory leading to quantitative prediction is also essential in seeking synergistic interventions that recognise the interplay between capacity, efficiency and resilience of soil, and to avoid the unintended consequences of land management that are directing us towards systemic collapse of productive land and an amenable climate.

## Methods

### Soils

We analysed soil from plots of the Rothamsted Highfield Ley-Arable field experiment (00:21:48° W, 51:48:18° N) set on soil that has been under permanent grass since at least 1838. The soil is a silty clay loam (25% clay: 62% silt: 13% sand) (Chromic Luvisol according to FAO criteria). In 2008, plots of severely degraded soil managed as bare fallow by regular tillage to remove any plants since 1959, were converted to arable and grassland managements. Arable soil was placed under continuous wheat rotation (winter wheat, *Triticum aestivum* L., currently cv. “Hereward” seed coated with Redigo Deter combination insecticide/fungicide treatment, Bayer CropScience) receiving ammonium nitrate fertilization to provide approximately 220 kg-N ha^−1^ annum^−1^, and additional 250 kg-K ha^−1^ and 65 kg-P ha^−1^ every three years, and grassland plots were maintained as a managed sward of mixed grasses and forbs. Plots were sampled annually, and soil was air-dried and sieved (< 2 mm) before being archived. To follow the development of soil structure in these soils, soil aggregates from continuous bare fallow, bare fallow converted to arable and bare fallow converted to grassland were selected for the years 2008, 2010, 2012, 2015 and 2018. In addition, we also sampled plots which had been managed consistently as bare fallow for fifty-two years, arable for sixty-two years or mixed grass swards since 1838. Physical and biological data has already been reported for these consistently managed soils (Table 1). Over these periods, the bare fallowed soils have become depleted in more labile organic carbon and enriched in persistent organic carbon^42^ and soil organic carbon has been reduced to a greater extent than in arable soil. There has also been an observable progressive shift, from grassland to arable and bare fallowed soil, in the distribution of organic carbon between different pools in the three soil managements, particularly a relative decline in discrete organic particles independent of stable soil aggregates, and a corresponding increase in the proportion of organic particles encapsulated in stable aggregates^23^. Confirmation of this apparent shift in soil structure has been provided by high-resolution X-ray Computed Tomography^25^.

### X-ray computed tomography and image analysis

We generated X-ray Computed Tomography (CT) images at 1.5 µm resolution and scales relevant to microbes (10^0^–10^2^ µm), requiring imaging of 0.7–2.0 mm diameter soil aggregates. The connectivity of pores within networks was assessed from binary images derived from X-ray CT using Minkowski functions^27^, basic geometric measures defined for binary structures. Aggregates were selected at random from soil collected from each plot of the Highfield experiment. Each was scanned using a Phoenix Nanotom system (GE Measurement and Control solution, Wunstorf, Germany) operated at 90 kV and a current of 65 µA. Initial image analysis was performed using Image-J. Images were threshold-adjusted using the bin bi-level approach of Vogel et al.^27^ using QuantIm version 4.01 (https://www.quantim.ufz.de/). Porosity and mean pore neck size were estimated directly from the threshold-adjusted binary images and Minkowski functions including Euler number—*χ*(*d*), pore size distribution, pore connectivity and surface area density were determined according to Vogel et al.^27^. *χ*(*d*) is a well-defined characteristic related to pore space topology and shown to be critical to hydraulic properties^43^. In three dimensions, *χ*(*d*) is defined as the number of isolated pores (of diameter, *d*) minus the number of redundant connections within the pore space, plus the number of enclosed pores^44^. Using this approach, we estimated Euler number density—*χ*(*d*)/*V*, where *V* represents the image volume—of the pore network of aggregates from each continuously managed Highfield soil.

### Calculation of diffusion in soil pore networks

The hierarchical soil structures revealed in X-ray CT images indicate that gaseous O_2_ in the atmosphere moves into soil primarily through its inter-aggregate pores and is then dissolved in water prior to moving into the aggregates largely by molecular diffusion. Since gaseous O_2_ diffuses up to 10^3^-fold more quickly than O_2_ dissolved in water, microbial community activity is thus constrained mainly by O_2_ diffusion within aggregates. The ability of aggregates to conduct dissolved O_2_ and other soluble substrates depends on the intra-aggregate pore geometry, and we quantified it with effective diffusion coefficients calculated directly by mimicking solute movement through the pore geometry using numerical simulations. The movement of solutes, including O_2_ and substrates, within the pore geometry is assumed to be diffusion dominated. For the images illustrated in Supplementary Fig. 9, the temporal change in solute concentration inside any pore voxel can be calculated using the finite volume approach, as follows:

1 $$\begin{gathered} \frac{{c_{o}^{t + \delta t} - c_{o}^{t} }}{\delta t} = q_{w} + q_{e} + q_{s} + q_{n} + q_{u} + q_{d} , \hfill \\ q_{w} = \left\{ {\begin{array}{*{20}l} {D(c_{w}^{t + \delta t} - c_{o}^{t + \delta t} )} & {if\,voxel\,w\,is\,pore} \\ {0,} & {if\,voxel\,w\,is\,solid} \\ \end{array} } \right.,\,\,q_{e} = \left\{ {\begin{array}{*{20}l} {D(c_{e}^{t + \delta t} - c_{o}^{t + \delta t} )} & {if\,voxel\,e\,is\,pore} \\ {0,} & {if\,voxel\,e\,is\,solid} \\ \end{array} } \right. \hfill \\ q_{s} = \left\{ {\begin{array}{*{20}l} {D(c_{n}^{t + \delta t} - c_{o}^{t + \delta t} )} & {if\,voxel\,s\,is\,pore} \\ {0,} & {if\,voxel\,s\,is\,solid} \\ \end{array} } \right.,\,\,q_{n} = \left\{ {\begin{array}{*{20}l} {D(c_{s}^{t + \delta t} - c_{o}^{t + \delta t} )} & {if\,voxel\,n\,is\,pore} \\ {0,} & {if\,voxel\,n\,is\,solid} \\ \end{array} } \right. \hfill \\ q_{d} = \left\{ {\begin{array}{*{20}l} {D(c_{d}^{t + \delta t} - c_{o}^{t + \delta t} )} & {if\,voxel\,d\,is\,pore} \\ {0,} & {if\,voxel\,d\,is\,solid} \\ \end{array} } \right.,\,\,q_{u} = \left\{ {\begin{array}{*{20}l} {D(c_{u}^{t + \delta t} - c_{o}^{t + \delta t} )} & {if\,voxel\,u\,is\,pore} \\ {0,} & {if\,voxel\,u\,is\,solid} \\ \end{array} } \right., \hfill \\ \end{gathered}$$

where *c* is concentration, *q* is diffusive flux, *D* is molecular diffusion of the solute in liquid water, superscripts *t* and *t* + δ*t* represent time, δ*t* is a time increment, subscript *o* represents the pore voxel being calculated, and subscripts *w*, *e*, *s*, *n*, *u* and *d* represents the face-to-face neighbours of voxel *o* on the west, east, south, north, top and bottom sides respectively. Applying Eq. (1) to all pore voxels leads to linear systems which was solved by the bi-conjugate gradient stabilized method^45^.

### Calculation of diffusion coefficients

To calculate the effective diffusion coefficient of each aggregate, we applied a constant concentration *C*_1_ on the top and a constant concentration *C*_o_ on the bottom of the image, and then simulated solute diffusion to steady state. The diffusive flux in the three directions in each pore voxel was calculated by Eq. 1. Taking the vertical direction as the *z* direction for the image, the effective diffusion coefficient of the image was calculated as follows:

2 $$D_{eff} = \frac{{L_{z} \sum\nolimits_{i = 1}^{N} {q_{z} (x_{i} )} }}{{N(C_{1} - C_{0} )}},$$

where *D*_*eff*_ is the effective diffusion coefficient, *N* is the total number of pore voxels in the simulated images, $q_{z} (x_{i} )$ is the vertical diffusive flux in pore voxel centred at location *x*_*i*_, *L*_*z*_ is the height of the image as shown in Supplementary Fig. 9. To address the impact of change in pore geometry due to management on the ability of the aggregate to diffuse solute, in result analysis we normalized the effective diffusion coefficient *D*_*eff*_ of all solutes by their associated molecular diffusion coefficient in non-constrained water, *D*.

### Modelling of oxygen diffusion and anoxia

The impact of soil structure on O_2_ diffusion and its subsequent consumption by microbes under various saturations was studied using pore-scale simulations. We first calculated the spatial distribution and connectedness of different pores and then determined water distributions in pores under different matric potentials (*ψ*_m_). We assumed the soil was initially saturated and then applied a negative pressure *p* at the bottom to drain water. We assumed the soil was essentially hydrophilic in that only pores whose associated capillary pressure *p*_c_, calculated by $p_{c} = \sigma /r$ with σ being water–air surface tension, is less than *p* and that they form clusters which stretch from the top to the bottom of the structure can be drained. Supplementary Fig. 10A shows an example illustrating water distribution in the structure calculated using the method described above when the saturation is 55%.

Once the water distribution was determined for a given *ψ*_m_, we treated the water–air interfaces inside the structure as a boundary at which gaseous O_2_ dissolves and then moves toward the solid–water interface to be reduced by microbial reactions. The partial pressure of gaseous O_2_ in the simulated structure was assumed to be constant. Movement of dissolved O_2_ in the liquid water was simulated using the following diffusion–reaction equation:

3 $$\begin{gathered} \frac{\partial c}{{\partial t}} = \nabla D\nabla c - s, \hfill \\ \left. c \right|_{{\Gamma_{aw} }} = c_{s} , \hfill \\ \end{gathered}$$

where *c* is the concentration of dissolved O_2_, *D* is the molecular diffusion coefficient of O_2_ in water, Γ_aw_ is the air–water interface, *s* is microbial consumption, *c*_s_ is the saturated dissolved O_2_ concentration at the water–air interface calculated from Henry’s law, $c_{s} = p_{o} /H$ in which *H* is the Henry constant and *p*_o_ is the partial pressure of the gaseous O_2_ inside the structure. Microbial consumption was assumed to occur in water-filled voxels adjacent to the water–solid wall and described by the following Monod kinetic equation:

4 $$s = m_{c} k_{0} \frac{\left[ C \right]}{{k_{C} + \left[ C \right]}}\frac{c}{{k_{O} + c}},$$

where *m*_c_ is microbial biomass, *k*_0_ is kinetic parameter, [*C*] is the concentration of dissolved carbon. Since we are interested in impact of soil structure on development of anaerobic sites, we simulated O_2_ diffusion and reduction to steady state. In all simulations, we normalized Eqs. (3) and (4) as follows

$$\begin{gathered} \frac{{\partial c^{\prime}}}{{\partial t^{\prime}}} = \nabla D^{\prime}\nabla c^{\prime} - s^{\prime}, \hfill \\ \left. {c^{\prime}} \right|_{{\Gamma_{aw} }} = 1, \hfill \\ s^{\prime} = k^{\prime}\frac{c}{{k_{o}^{^{\prime}} + c}}, \hfill \\ \end{gathered}$$

where $t^{\prime} = t/T_{0}$, $D^{\prime} = DT_{0} /L^{2}$, $c^{\prime} = c/c_{s}$ and $k^{\prime} = m_{c} k_{0} T_{0} \left[ C \right]/\left( {k_{c} + [C]} \right)$ in which *L* is the side length of the voxels and *T*_0_ is a characteristic chosen to make $D^{\prime} = 1$ in our simulations.

The above equation was solved by a finite volume method with each water-filled voxel being the element used to calculate the mass balance. In all simulations, water was assumed to be initially free of O_2_ and we simulated the system to steady state. As the development of anaerobic areas was a balance between the ability of soil to diffuse dissolved O_2_ and the microbial consumption rate, to elucidate that the relative anaerobicity of soils under the same *ψ*_m_ is the consequence of their structures and does not change with microbial activity rate, we simulated two scenarios: a fast microbial decomposition (*k*′ = 1 × 10^–2^) and a slow microbial decomposition (*k*′ = 1 × 10^–4^). For each scenario, once the system was deemed to have reached a steady state, we sampled sites and where the concentration of dimension-less dissolved O_2_ was less than 20% assumed them to be anaerobic^46^. Supplementary Fig. 10B shows an illustrative example of the location of anaerobic areas simulated by the above method in which soil particles were made transparent. We repeated the procedure to achieve different water distributions calculated by varying *ψ*_m_ and then calculated the proportional change in the volumetric anaerobic sites with the *ψ*_m_ for both the fast and slow microbial reactions. The results are shown in Fig. 4 for soil samples taken from all treatments.

### Modelling of organic carbon dynamics in soil

We used RothC-26.3^47^ to model the turnover of soil C_org_ in the experimental soils, accounting for the effects of soil type, plant cover and historical temperature and moisture content on organic carbon turnover processes. We used the same inputs of C_org_ to the soil as those used by Johnston et al.^48^. To obtain the starting soil C_org_ of 63.6 Mg-C ha^−1^, input to the soil from plant debris, roots, and root exudates was 2.7 Mg-C ha^−1^, with inert organic matter (IOM) being 3.0 Mg-C ha^−1^. The incoming C_org_ from plant residues were assumed to have decomposable plant material (DPM) and resistant plant material (RPM) in the proportion 0.59 and 0.41, respectively; these are the default proportions for arable cropping and managed grassland. For the first 12 years after the experiment started, grass was grazed by sheep before management changed to a grass/clover sward, harvested once or twice a year for conservation. To reflect this, the modelled grass management received inputs of 5 Mg-C ha^−1^ annum^−1^ between 1949 and 1960, or 4 Mg-C ha^−1^ annum^−1^ between 1961 and 2016. Arable management received a carbon input of 1.4 Mg-C ha^−1^ annum^−1^ and bare fallow management received no inputs of carbon to the soil.

### DNA extraction and metagenome sequencing

Soil was collected from triplicate plots for each management to a depth of 10 cm using a 3-cm diameter corer. The top 2-cm of soil containing root mats and other plant detritus was discarded. Ten cores per plot were pooled and thoroughly mixed whilst sieving through a 2-mm mesh; samples were then frozen at − 80 °C. All implements were cleaned with 70% ethanol between sampling/sieving soil from each plot. Soil community DNA was extracted from a minimum of 2 g soil using the MoBio PowerSoil DNA isolation kit (Mo Bio Laboratories, Inc. Carlsbad, CA, USA) with three replicates for each soil treatment. When necessary, extracts from individual replicates were pooled to provide sufficient material for sequencing. 10 µg of high-quality DNA was provided for sequencing for each of the nine continuous management plots. Shotgun metagenomic sequencing of DNA was provided by Illumina (Great Abington, UK) using a HiSeq 2000 sequencing platform, generating 150-base, paired-end reads. The generated sequences were limited to a minimum quality score of 25 and a minimum read length of 70-bases using Trimmomatic^49^. After filtering to remove substandard sequences, the average metagenome size for each soil was 4.96 × 10^8^ reads for grassland, 2.86 × 10^8^ for arable and 2.88 × 10^8^ for bare fallow soils. Since differences in library sizes were less than tenfold, we did not employ rarefaction before comparing the datasets^50^.

### Bioinformatical analysis of metagenome sequences

To assess general abundance of taxa and genes in metagenomes, we mapped individual metagenomic sequences to the RefSeq non-redundant (NR) protein database held at NCBI (downloaded August 22nd, 2018) using DIAMOND version 0.8.27^51^ in BLASTX mode using a bitscore cut-off of 55. For each sequence, only the match with the highest bitscore was considered. Sequences not matching the NR database were considered currently unclassified. MEGAN Ultimate version 6.10.2^52^ was used to associate metagenome sequences with both taxa and Kyoto Encyclopaedia of Genes and Genomes (KEGG) functional orthologs and modules^53^. For taxa, MEGAN was used to establish Prokaryotic and Fungal community assemblages and calculate weighted UniFrac distances^54^ between assemblages associated with each land management. To identify the most diagnostic microorganisms characterising communities of each soil, we used supervised Random Forests (RF), a classification algorithm approach based upon a collection of unpruned decision trees^55^, each built using a bootstrap sample of training data using a randomly selected subset of OTUs. The RF classifier was built by growing 5,000 classification trees. The prediction performance and confusion matrices were determined using out-of-bag cross-validation. The percent mean decrease in accuracy of the importance matrix was used to select taxa that were most predictive of each microbiome assemblage. RF was employed as implemented in MicrobiomeAnalyst^56^. In addition, bacterial communities were also compared based upon the abundance and phylogenetic relatedness of metagenome reads homologous to the bacterial 16S rRNA gene. A 16S rRNA profile hidden Markov model (pHMM) was generated based upon an alignment of the set of 4,528 reference sequences associated with paprica^57^, built December 2017. Metagenome reads with homology to the 16S rRNA pHMM were identified using hmmsearch^58^ with a 1 × 10^–5^ Expect-value (*E*) cut-off and assigned to branches of the fixed maximum likelihood 16S rRNA phylogenetic tree using a phylogenetic placement algorithm, pplacer version 1.1alpha10^59^. To assess 16S rRNA gene-based β-diversity in the different soils, Kantorovich-Rubinstein (KR) phylogenetic distance metrics^60^ were calculated from phylogenetic placements of metagenome reads using the guppy kr binary (part of the pplacer suite), treating each query as a point mass concentrated on the highest-weight placement. The advantage of the KR distance metric is that it compares gene assemblage distributions on a phylogenetic tree (of 16S rRNA or other genes), in units of nucleotide substitutions per site, and is therefore a biologically meaningful approach to comparing communities.

From all of the reads binned to a KEGG orthologous group, we selected those associated with carbohydrate metabolism (ko09101) [including glycolysis/gluconeogenesis (ko00010), citrate cycle (ko00020), pentose phosphate pathway (ko00030), pentose and glucuronate interconversions (ko00040), fructose and mannose metabolism (ko00051), galactose metabolism (ko00052), ascorbate and aldarate metabolism (ko00053), starch and sucrose metabolism (ko00500), amino sugar and nucleotide sugar metabolism (ko00520), pyruvate metabolism (ko00620), glyoxylate and dicarboxylate metabolism (ko00630), propanoate metabolism (ko00640), butanoate metabolism (ko00650), C5-branched dibasic acid metabolism (ko00660), inositol phosphate metabolism (ko00562)], methane metabolism (ko00680), carbon fixation pathways in prokaryotes (ko00720), nitrogen metabolism (ko00910), sulfur metabolism (ko00920), xenobiotics biodegradation and metabolism (ko09111) [including benzoate degradation (ko00362), aminobenzoate degradation (ko00627), fluorobenzoate degradation (ko00364), chloroalkane and chloroalkene degradation (ko00625), chlorocyclohexane and chlorobenzene degradation (ko00361), toluene degradation (ko00623), xylene degradation (ko00622), nitrotoluene degradation (ko00633), ethylbenzene degradation (ko00642), styrene degradation (ko00643), atrazine degradation (ko00791), caprolactam degradation (ko00930), dioxin degradation (ko00621), naphthalene degradation (ko00626), polycyclic aromatic hydrocarbon degradation (ko00624), furfural degradation (ko00365), steroid degradation (ko00984), metabolism of xenobiotics by cytochrome P450 (ko00980) and drug metabolism—other enzymes (ko00983)], enzyme families (ko09112), membrane transport (ko09131) [including transporters (ko02000), ABC transporters (ko02010), phosphotransferase systems (ko02060), bacterial secretion systems (ko03070) and secretion systems (ko02044)], two-component systems (ko02020 and 02022), biofilm formation—*Vibrio cholerae* (ko05111), —*Pseudomonas aeruginosa* (ko02025), —*Escherichia coli* (ko02026), bacterial chemotaxis (ko02030), bacterial motility proteins (ko02035), and flagellar assembly (ko02040) for detailed study of abundance differences between the soils. Where necessary, KEGG orthologs were associated with higher-order functions by mapping to the KEGG BRITE functional hierarchy classification. In total, 8,857 KEGG functional orthologs were identified. To identify genes for which a significant difference in abundance between the treatments was observed we used DESeq2^61^ which employs a negative binomial generalized linear model to generate maximum-likelihood estimates for the log_2_-fold change between conditions associated with each gene. Bayesian shrinkage, based upon a zero-centred normal distribution as a prior, reduces the log_2_-fold change towards zero for genes with low mean counts or a high dispersion in their count distribution. The resulting shrunken fold-changes are used in tests of significance using Wald’s test. DESeq2 has been shown to be particularly sensitive to differences in gene abundance on small datasets^50^ such as those in this study. Before analysis, 3,930 low abundance genes were removed (minimum mean count of 20) as well as 986 genes with the lowest coefficients of variation. Differential abundance of the remaining 3,940 genes was tested for significance employing α = 0.05 and a Benjamini–Hochberg false discovery rate (*q*) of 0.1 to control type I error rate in the face of multiple comparisons. DESeq2 was employed as implemented in MicrobiomeAnalyst.

### Statistical analysis

One-factor analysis of variance was employed to test the effect of soil treatment upon *d*_crit_ and modelled diffusion coefficients arising from X-ray CT, and phylogenetic diversity estimates of α-diversity arising from metagenomic analysis. Where a significant treatment effect was observed, post hoc pairwise comparisons were performed using Tukey’s HSD test (*Q*) employing the Copenhaver and Holland multiple comparisons procedure. These tests were performed in PAST version 3.25^62^. One-factor analysis of covariance (ANCOVA) was used to test for treatment effects upon the formation of connected porosity in degraded soil following conversion to either arable or grassland, using time post conversion as the covariate. The assumption of homogeneity of slopes was tested first, before ANCOVA was used to test for treatment effects using an equal slopes model. Post hoc Holm–Šidák multiple pair-wise comparisons were used to establish whether differences in adjusted mean connected porosity between treatments were significant. ANCOVA was performed in SigmaPlot for Windows version 14.0 (Systat Software Inc., San Jose, CA, USA).

For metagenome-associated multivariate data, we first compared prokaryotic and fungal communities by calculating unrooted phylogenetic Neighbour-Nets^63^ using weighted UniFrac distances and compared the 16S rRNA-conditional bacterial assemblages using KR distances. KR distance-based analyses were performed after testing for heteroscedasticity using the PERMDISP test^64^. Hypothesis testing was based upon permutational multivariate analysis of variance^65^ (PERMANOVA) and post hoc pair-wise tests. Differences between treatment were visualized using Principal Coordinates Analysis using KR distance. To identify associations between chemical and physical edaphic factors and any treatment effects, distance-based linear modelling^66^ was used to identify the best combination of edaphic factors to model the multivariate data; the resulting model was visualized using distance-based redundancy analysis. All multivariate tests were performed in PRIMER PERMANOVA + version 7.0.13 (PRIMER-e, Auckland, New Zealand) and probabilities were based upon 99,999 permutations (denoted *p*_perm_). For post hoc pair-wise comparisons, since the number of observations was insufficient to allow a reasonable number of permutations, Monte Carlo probabilities (denoted *p*_MC_) were calculated based upon an asymptotic permutation distribution^67^.

## Supplementary information

Supplementary information

## Data Availability

Data, code, and materials described in this research are available together with extensive chemical, climate and treatment data and history on the *e*-RA database, maintained by Rothamsted Research.
