# Supplementary material for: Soil as an extended composite phenotype of the microbial metagenome
Source: Sci Rep. 2020 Jun 30;10:10649. doi: 10.1038/s41598-020-67631-0 (PMC7327058; doi:10.1038/s41598-020-67631-0)
Supplement: Supplementary file 1 — Supplementary information [file 41598_2020_67631_MOESM1_ESM.docx]

| *Scientific Reports* |
| --- |
| Soil as an Extended Composite Phenotype of the Microbial Metagenome. |
| Supplementary Figures |

| Andrew L. Neal, Aurélie Bacq-Labreuil, Xiaoxian Zhang, Ian M. Clark, Kevin Coleman, Sacha J. Mooney, Karl Ritz, John W. Crawford |
| --- |


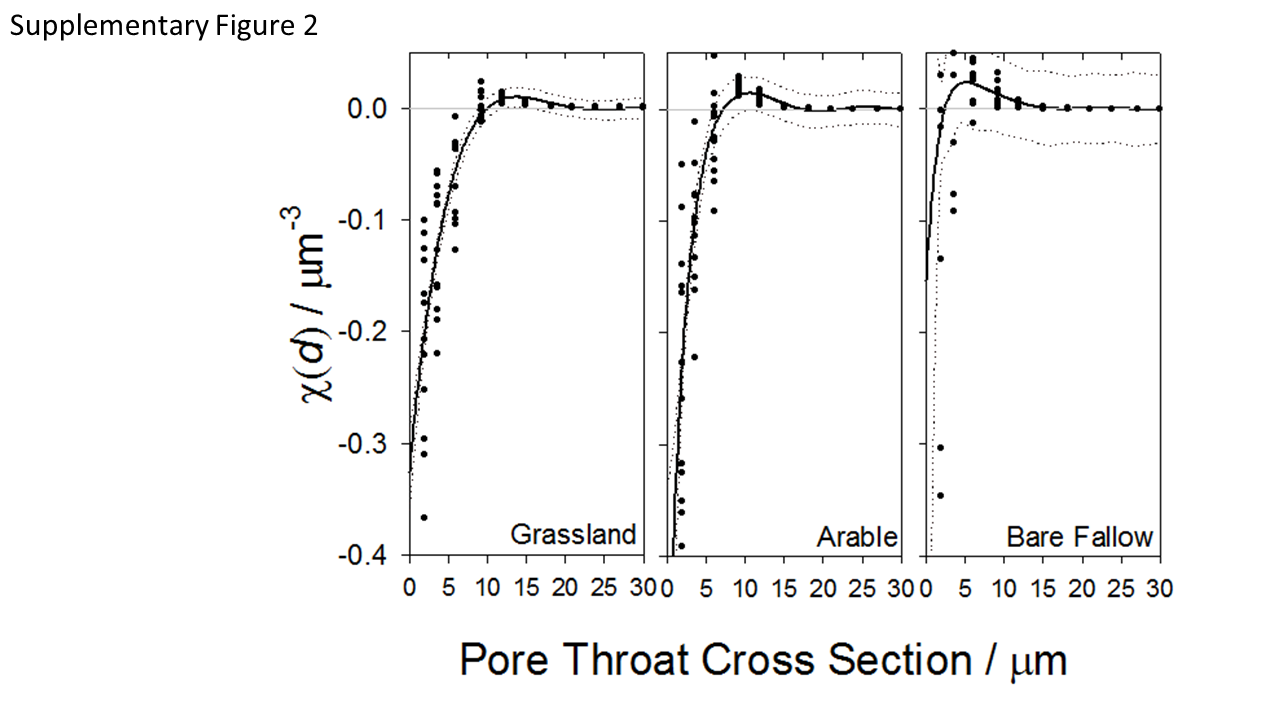


**Supplementary Figure 1. Euler connectivity function curves for Highfield soils generated from high-resolution (1.5 µm) X-ray Computed Tomography.** Each curve presents the connectivity within and between different pore size classes. For connected pores, χ(*d*) takes negative values and unconnected pores positive values. The pore diameter where χ(*d*) = 0 was estimated by fitting a polynomial to the combined data from three representative aggregates for each soil. This value, designated *d*_crit_, was used as a descriptor of pore connectivity to establish the relationship between soil physical structure and differences in taxonomy and function established from metagenomics. For each soil, the solid line represents the polynomial fit to the combined data, the dashed curves represent the upper and lower 95% confidence intervals of the fit.


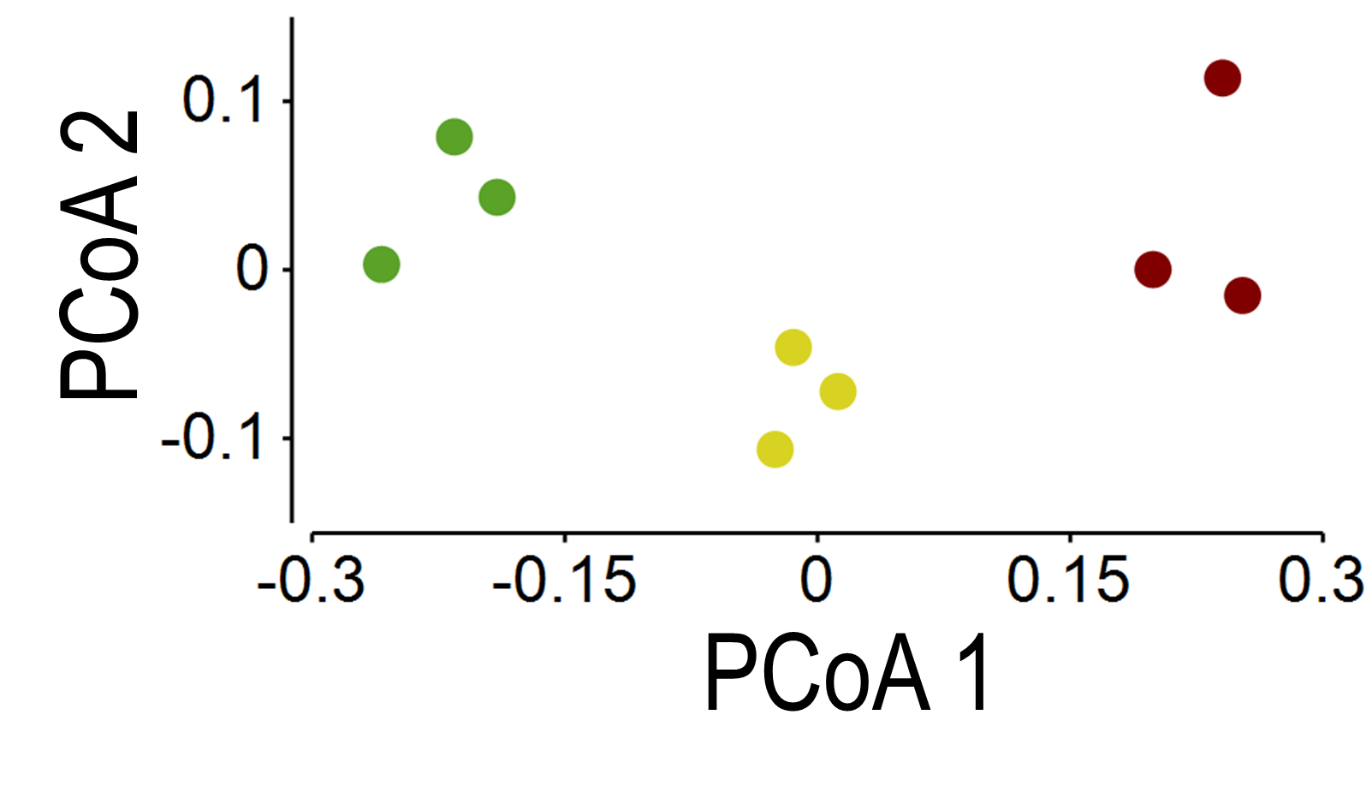


**Supplementary Figure 2. Unconstrained management-conditional principal coordinate analysis of bacterial assemblages associated with consistently managed plots of the Highfield Ley-Arable long-term field experiment.** Ordination is based upon Kantorovich-Rubinstein distances calculated from placement of homologous metagenome reads on the bacterial 16S rRNA gene reference phylogenetic tree. Data points represent individual replicate plots of grassland (green), arable (yellow) and bare fallow (brown) soils. PCoA axis 1 (eigenvalue = 0.312) accounted for 79.03% of total variability, PCoA axis 2 (eigenvalue = 0.040) accounted for 10.12% of total variation.


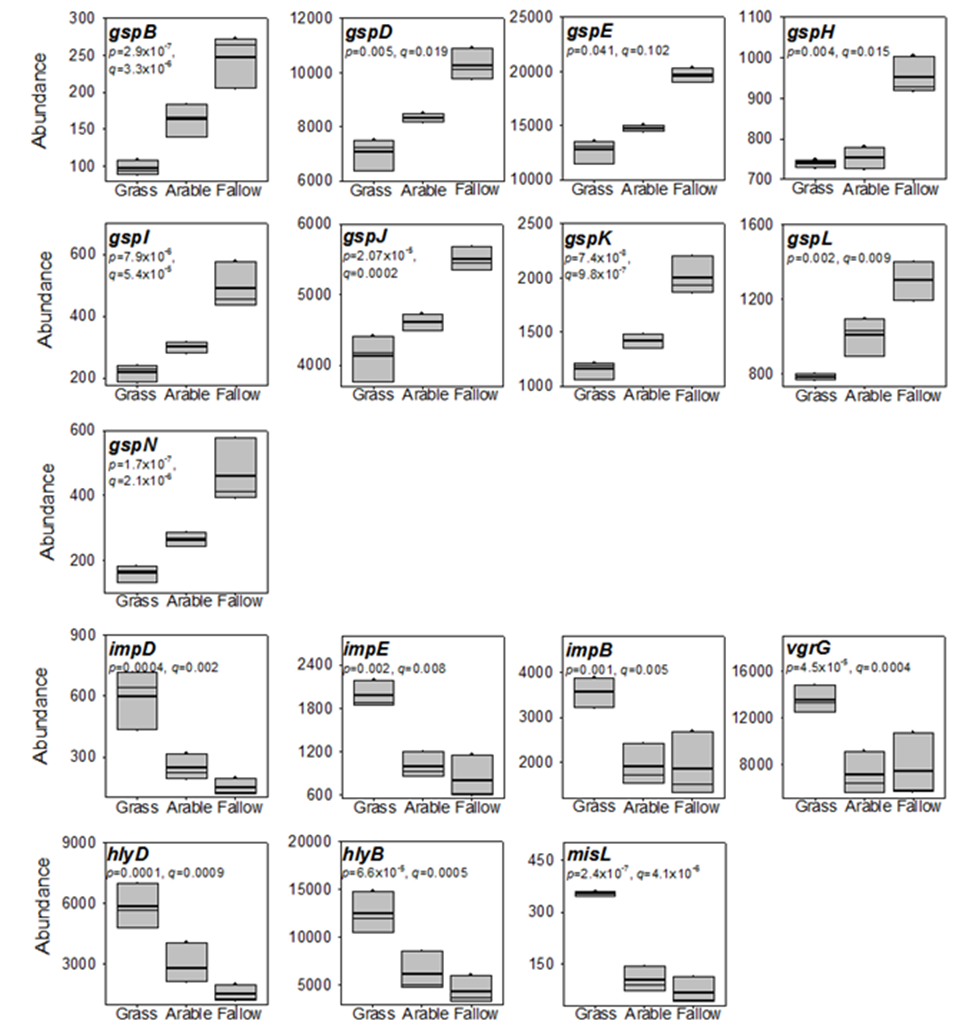


**Supplementary Figure 3. Genes associated with protein secretion in bacteria are more abundant in bare fallow soil.** Box plot of abundance for genes associated with the Type II Secretion System (*gspB* – *N*), the Type VI Secretion System (*impDEB* and *vgrG*), the Type I Secretion System (*hlyD*, *hlyB*) and the Type V Secretion System (*misL*) under different land managements. Box plot shows the mean (bold line) and median (light line) abundance together with the 5^th^ and 95^th^ percentiles. The significance (*p*) and positive false discovery rate (*q*) of the difference in abundance between the three treatments are shown.


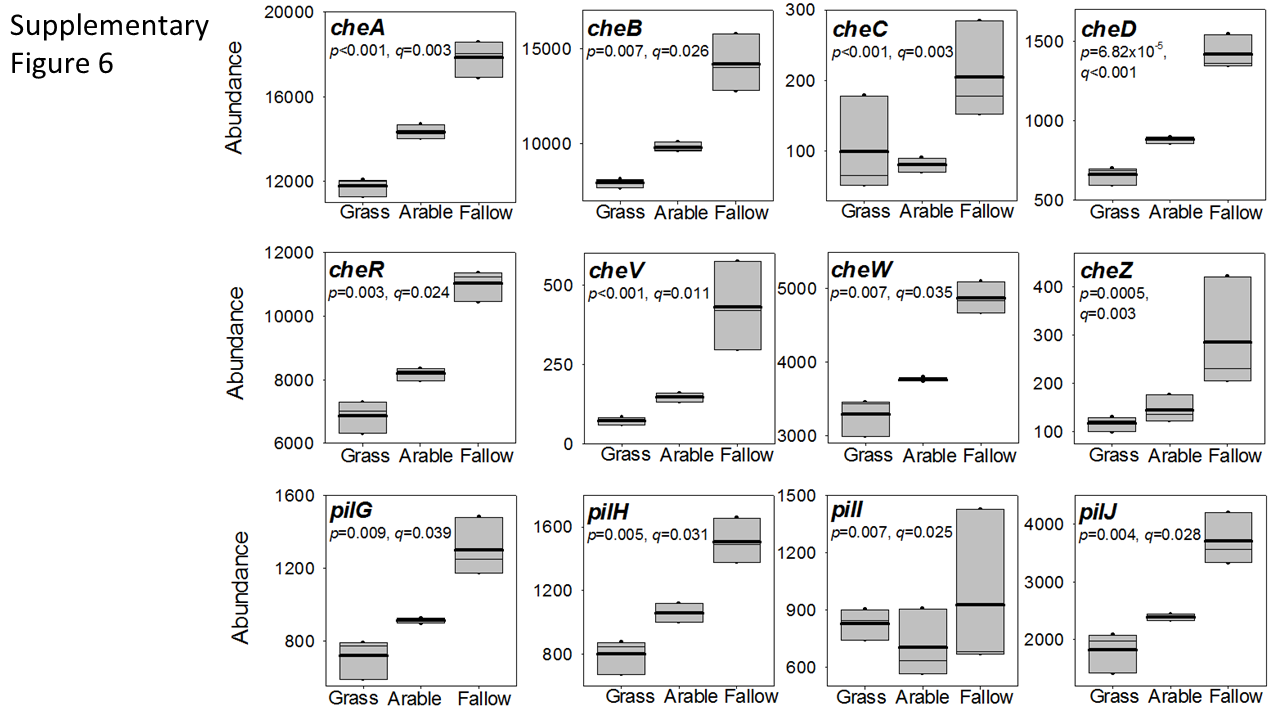


**Supplementary Figure 4. Genes associated with chemotaxis and motility in bacteria are more abundant in Bare fallow soil.** Box plot of abundance for genes associated with chemotaxis (*cheA* – *Z*), type IV pili synthesis (*pilG* – *J*) under different land managements. Box plot shows the mean (bold line) and median (light line) abundance together with the 5^th^ and 95^th^ percentiles. The significance (*p*) and positive false discovery rate (*q*) of the difference in abundance between the three treatments are shown.


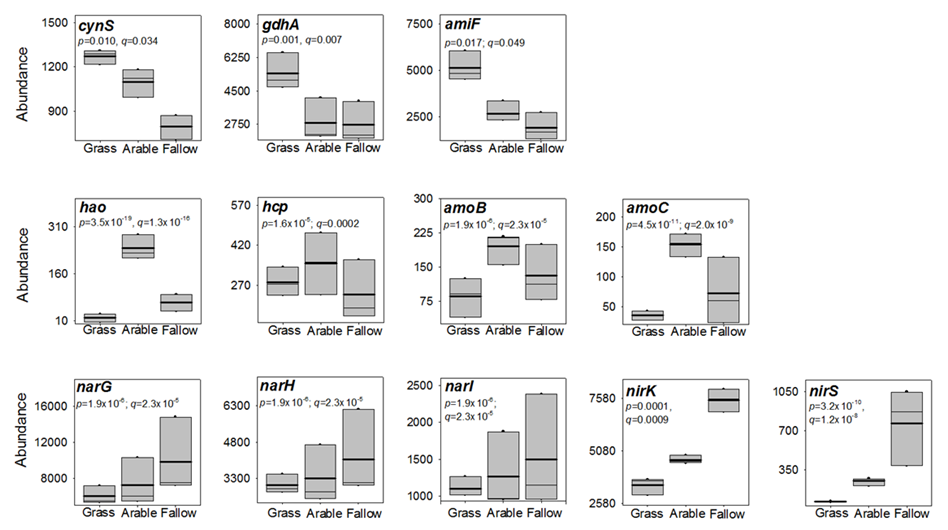


**Supplementary Figure 5. Genes associated with nitrogen metabolism show land-use specific responses.** Box plot of abundance for genes associated with cyanate lyase (*cynS*), glutamate dehydrogenase (*gdhA*), the oligopeptide ABC transporter, ATP-binding protein (*amiF*), hydroxylamine dehydrogenase (*hao*), hydroxylamine reductase (*hcp*), the methane/ammonia monooxygenase subunits B and C (*amoB*, *amoC*), nitrate reductase/nitrite oxidoreductase alpha- beta- and gamma-subunits (*narGHI*), and nitrite reductase (*nirK* and *nirS*). Box plot shows the mean (bold line) and median (light line) abundance together with the 5^th^ and 95^th^ percentiles. The significance (*p*) and positive false discovery rate (*q*) of the difference in abundance between the three treatments are shown.


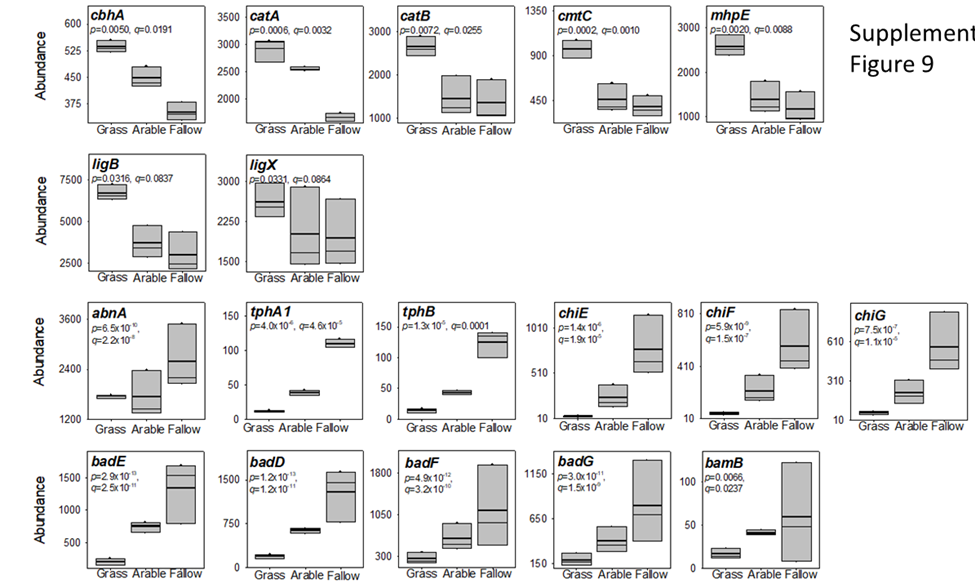


**Supplementary Figure 6. Genes associated with carbohydrate metabolism show land-use specific responses.** Box plot of abundance for genes associated with cellulose 1,4-β-cellobiosidase (*cbhA*), catechol 1,2-dioxygenase (*catA*), muconate cycloisomerase (*catB*), 2,3-dihydroxy-*p*-cumate/2,3-dihydroxybenzoate-3,4-dioxygenase (*cmtC*), 4-hydroxy-2-oxovalerate aldolase (*mhpE*), protocatechuate 4,5-dioxygenase (*ligB*), 5,5'-dehydrodivanillate *O*-demethylase (*ligX*), arabinan endo-1,5-α-*L*-arabinosidase (*abnA*), terephthalate 1,2-dioxygenase reductase (*tphA1*), 1,2-dihydroxy-3,5-cyclohexadiene-1,4-dicarboxylate dehydrogenase (*tphB*), GH family 18 chitinase *chiE*, and GH family 19 chitinases *chiF* and *chiG*, benzoyl-CoA reductase subunits A, B, C and D (*badFEDG*) and benzoyl-CoA reductase subunit (*bamB*). Box plot shows the mean (bold line) and median (light line) abundance together with the 5^th^ and 95^th^ percentiles. The significance (*p*) and positive false discovery rate (*q*) of the difference in abundance between the three treatments are shown.


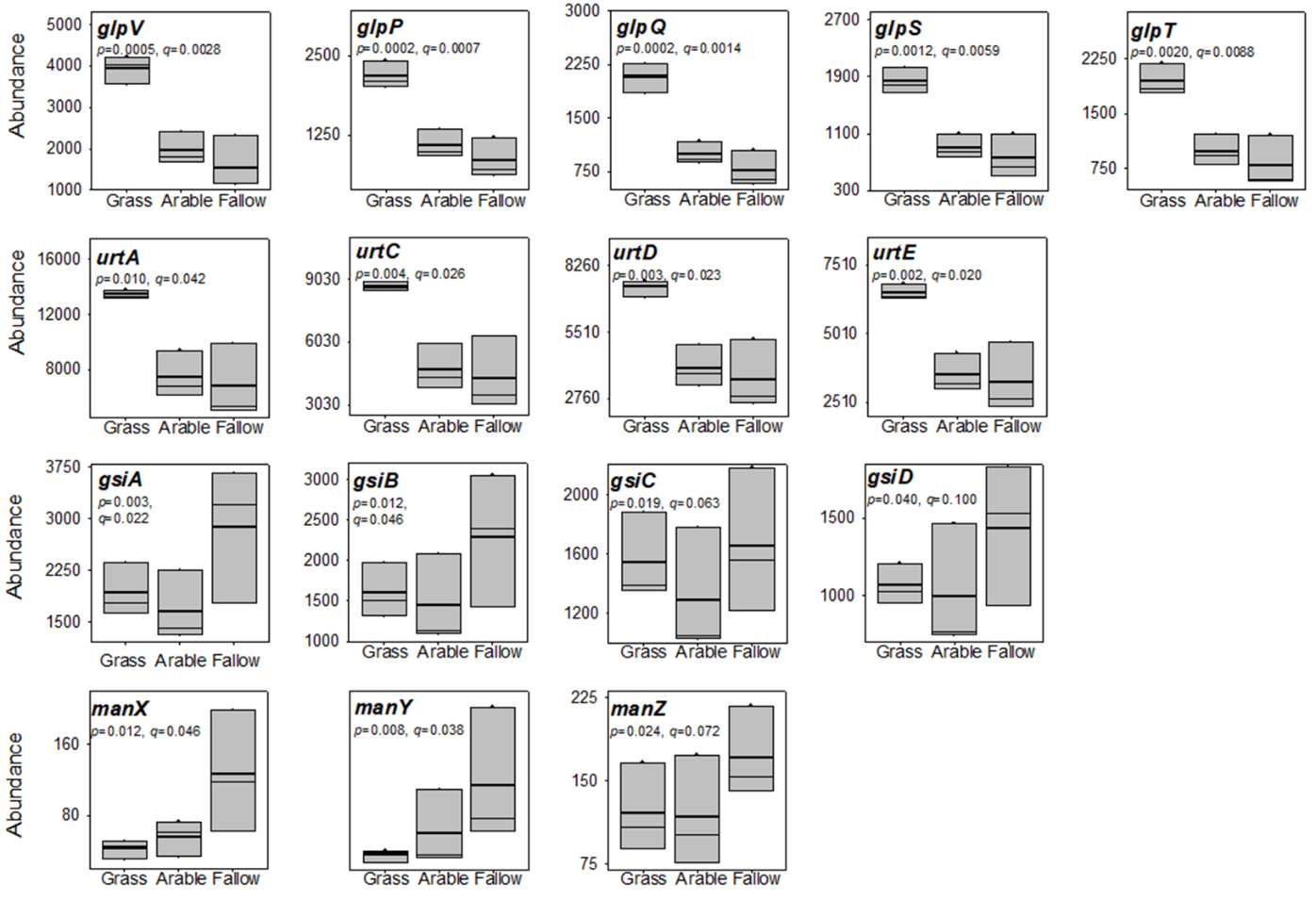


**Supplementary Figure 7. Genes associated with solute transport show land-use specific responses.** Box plot of abundance for genes associated with transport of glycerol (*glpVPQST*), urea (*urtACDE*), glutathione (*gsiABCD*) and glucose, mannose, glucosamine and *N*-acetylglucosamine transport (*manXYZ*). Box plot shows the mean (bold line) and median (light line) abundance together with the 5^th^ and 95^th^ percentiles. The significance (*p*) and positive false discovery rate (*q*) of the difference in abundance between the three treatments are shown.


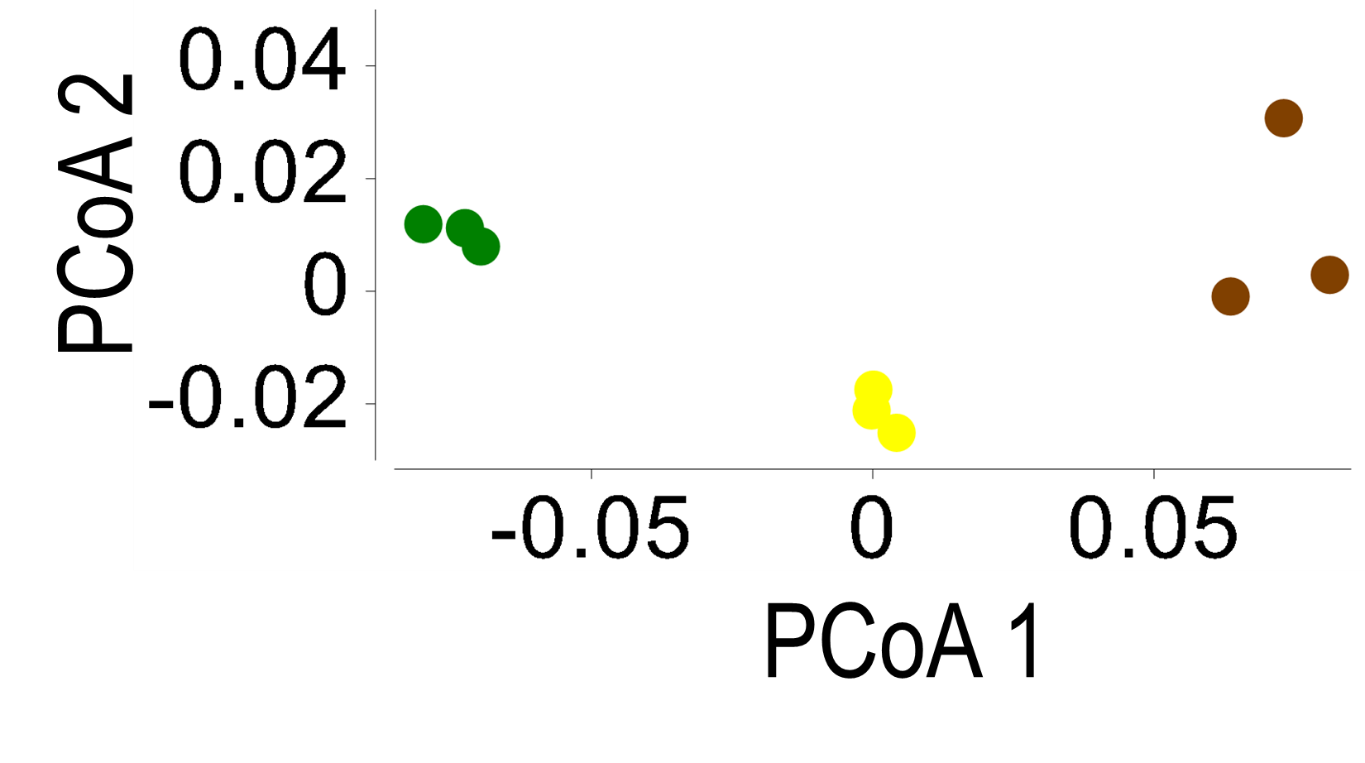


**Supplementary Figure 8. Unconstrained management-conditional principal coordinate analysis of gene assemblages associated with consistently managed plots of the Highfield Ley-Arable long-term field experiment.** Ordination is based on Hellinger distances calculated from square root transformed gene abundance data. Data points represent individual replicate plots of grassland (green), arable (yellow) and bare fallow (brown) soils. PCoA axis 1 (eigenvalue = 0.032) accounted for 84.48% of total variability, PCoA axis 2 (eigenvalue = 0.0027) accounted for 6.94% of total variation.


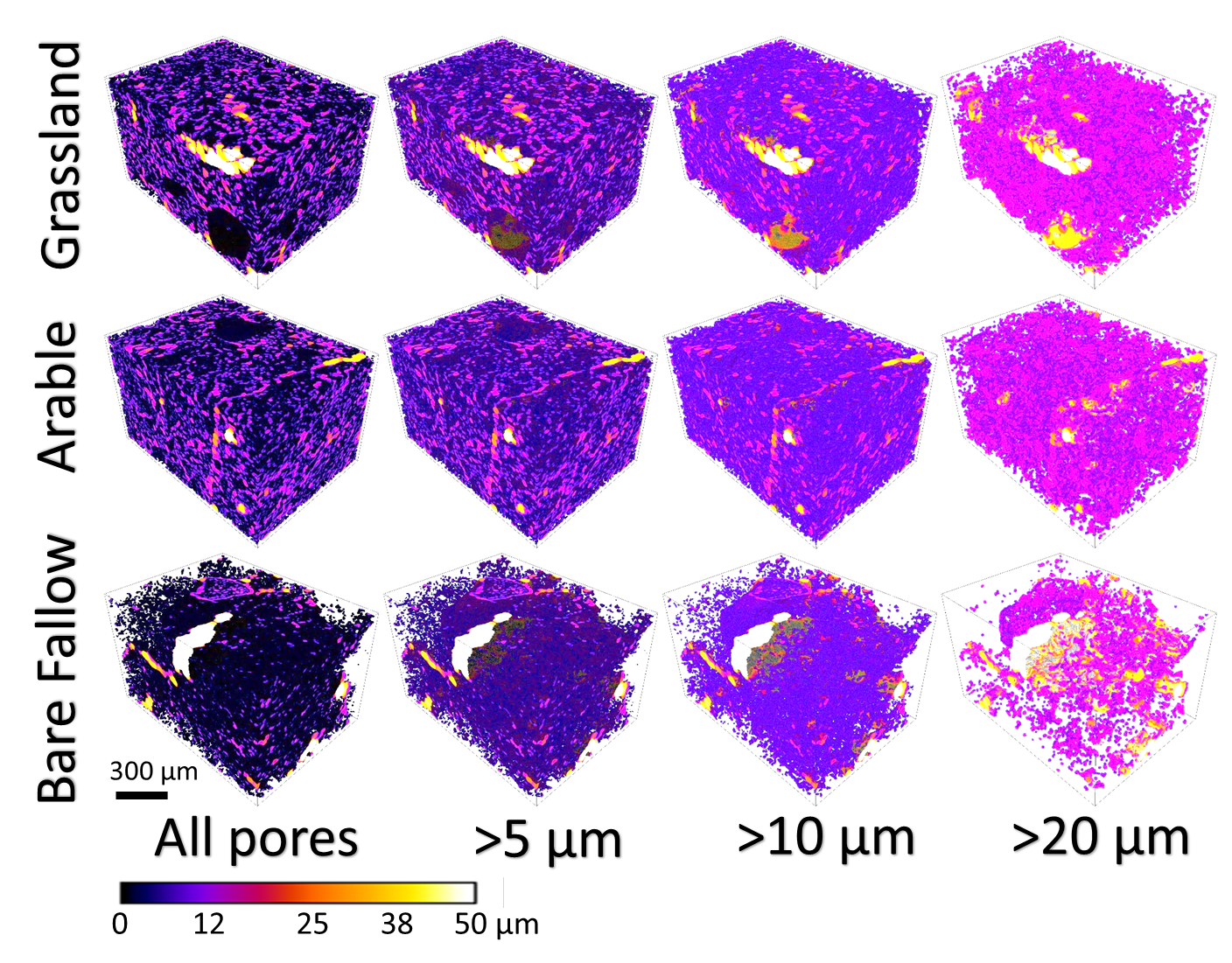


**Supplementary Figure 9. Three-dimensional representation of soil porosity in Highfield soils.** Soil structures were determined from high-resolution (1.5 µm) X-ray Computed Tomography of aggregates (<2 mm) collected from long-term grassland, arable and bare fallowed soils. The images are pseudo-coloured to reflect the ranges of pore throat diameters present in each soil (scale shown below images) and are shown at increasingly larger pore throat diameter cut-offs for ease of discrimination. Each representation is of a typical aggregate structure collected from each soil.


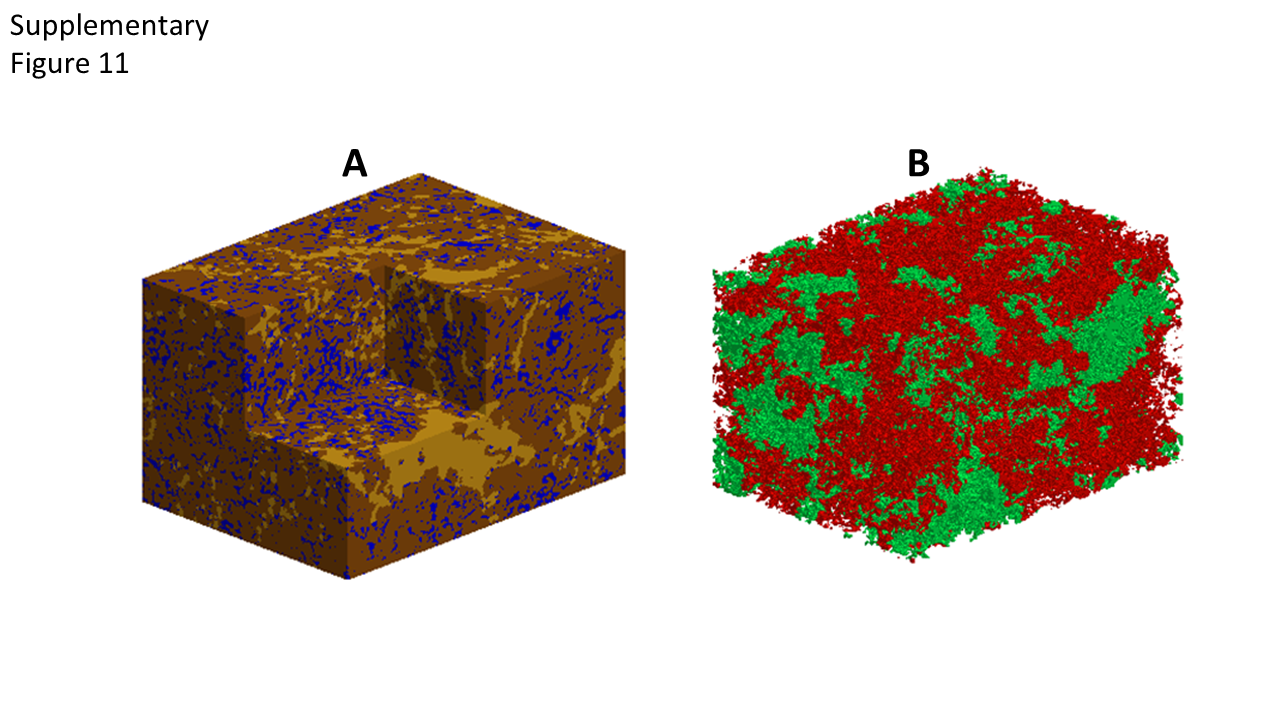


**Supplementary Figure 10.** Water distribution and anoxic pore space within soils. A - An illustrative example showing the distribution of water (blue), air (yellow) and soil particles (brown) at saturation of 55%, calculated using the described method. B - Location of anoxic (green) and aerobic (red) areas calculated from the pore-scale simulation after the system reaches steady state for *k*’ = 5x10^-3^.
